# Active Anaerobic Archaeal Methanotrophs in Recently Emerged Cold Seeps of Northern South China Sea

**DOI:** 10.3389/fmicb.2020.612135

**Published:** 2020-12-16

**Authors:** Tingting Zhang, Xi Xiao, Songze Chen, Jing Zhao, Zongheng Chen, Junxi Feng, Qianyong Liang, Tommy J. Phelps, Chuanlun Zhang

**Affiliations:** ^1^Guangzhou Marine Geological Survey, China Geological Survey, Guangzhou, China; ^2^Gas Hydrate Engineering Technology Center, China Geological Survey, Guangzhou, China; ^3^Southern Marine Science and Engineering Guangdong Laboratory (Guangzhou), Guangzhou, China; ^4^Department of Ocean Science and Engineering, Southern University of Science and Technology, Shenzhen, China; ^5^Shenzhen Key Laboratory of Marine Archaea Geo-Omics, Southern University of Science and Technology, Shenzhen, China; ^6^Earth and Planetary Sciences, University of Tennessee, Knoxville, Knoxville, TN, United States

**Keywords:** anaerobic methanotrophs, methane, anaerobic oxidation of methane, cold seeps, calyptogena

## Abstract

Cold seep ecosystems are developed from methane-rich fluids in organic rich continental slopes, which are the source of various dense microbial and faunal populations. Extensive studies have been conducted on microbial populations in this unique environment; most of them were based on DNA, which could not resolve the activity of extant organisms. In this study, RNA and DNA analyses were performed to evaluate the active archaeal and bacterial communities and their network correlations, particularly those participating in the methane cycle at three sites of newly developed cold seeps in the northern South China Sea (nSCS). The results showed that both archaeal and bacterial communities were significantly different at the RNA and DNA levels, revealing a higher abundance of methane-metabolizing archaea and sulfate-reducing bacteria in RNA sequencing libraries. Site ROV07-01, which exhibited extensive accumulation of deceased *Calyptogena* clam shells, was highly developed, and showed diverse and active anaerobic archaeal methanotrophs (ANME)-2a/b and sulfate-reducing bacteria from RNA libraries. Site ROV07-02, located near carbonate crusts with few clam shell debris, appeared to be poorly developed, less anaerobic and less active. Site ROV05-02, colonized by living *Calyptogena* clams, could likely be intermediary between ROV07-01 and ROV07-02, showing abundant ANME-2dI and sulfate-reducing bacteria in RNA libraries. The high-proportions of ANME-2dI, with respect to ANME-2dII in the site ROV07-01 was the first report from nSCS, which could be associated with recently developed cold seeps. Both ANME-2dI and ANME-2a/b showed close networked relationships with sulfate-reducing bacteria; however, they were not associated with the same microbial operational taxonomic units (OTUs). Based on the geochemical gradients and the megafaunal settlements as well as the niche specificities and syntrophic relationships, ANMEs appeared to change in community structure with the evolution of cold seeps, which may be associated with the heterogeneity of their geochemical processes. This study enriched our understanding of more active sulfate-dependent anaerobic oxidation of methane (AOM) in poorly developed and active cold seep sediments by contrasting DNA- and RNA-derived community structure and activity indicators.

## Introduction

Methane is a potent greenhouse gas. It is 28 times more efficient than CO_2_ and contributes more than 20% to global warming (IPCC, 2014). From 2014 to the end of 2018 atmospheric methane increased at twice the rate observed in 2007 (Fletcher and Schaefer, 2019). The exact reason for the increasing methane in the atmosphere remains unclear. Therefore, studying global methane oxidation process is becoming increasingly important.

Continental margins account for only 11% of the ocean area (Levin and Sibuet, 2012); however, their subsurface seabed contains large reservoirs of methane in the dissolved state, gaseous state, and in the form of solid natural gas hydrates. Driven by a variety of unstable geological factors, low-temperature and methane-rich fluids emit to the seabed surface along the seabed channels, forming a unique deep-sea ecosystem—cold seep (Boetius and Wenzhöfer, 2013). There are thousands of active cold-seep systems distributed on the continental margins around the world. Although they emit 0.01–0.05 Gt of carbon up to the atmosphere annually (Milkov et al., 2003; Kvenvolden and Rogers, 2005; Reeburgh, 2007; Judd and Hovland, 2009), large amounts of methane have been consumed by microbial oxidation during upward migration in sediments (Reeburgh, 2007; Regnier et al., 2011). Therefore, methane oxidation by microorganisms in sediments plays an important role in preventing cold seep methane from methane from entering the atmosphere (Knittel and Boetius, 2009; Boetius and Wenzhöfer, 2013).

Anaerobic archaeal methanotrophs (ANMEs) compose the major microbial groups in cold seep sediments, with a relative abundance up to 80–90% in total archaea (Vigneron et al., 2013; Cui et al., 2019). They can be divided into three distinct methanotrophic groups, namely ANME-1, ANME-2, and ANME-3. The large phylogenetic distance between ANME groups represents their internal sequence similarity of 75–92% according to 16S rRNA genes (Knittel and Boetius, 2009). ANME-1 is further divided into two subgroups, designated ANME-1a and ANME-1b (Knittel et al., 2005). ANME-2 is the most widely distributed and highly diverse group, which is divided into four distinct subgroups, ANME-2a, ANME-2b, ANME-2c, and ANME-2d (Orphan et al., 2001; Mills et al., 2003). ANME-2a/b is highly abundant in cold seep sediments (Knittel et al., 2005; Niu et al., 2017), and ANME-2c can be found in both hydrothermal and cold seep sediments (Vigneron et al., 2013; McKay et al., 2016). ANME-2d is also named “GOM Arc I” (Lloyd et al., 2006) due to its late discovery and distant relationship with other subgroups of ANME-2. “*Candidatus* Methanoperedens nitroreducens” has also been used to refer to ANME-2d due to its capability of performing AOM with nitrate as the ultimate electron acceptor (Haroon et al., 2013).

ANMEs frequently form consortia with sulfate-reducing bacteria to conduct anaerobic oxidation of methane (AOM). ANME-1 and ANME-2 are connected with *Desulfosarcina*–*Desulfococcus* (DSS) as sulfate-reducing bacteria (Orphan et al., 2002), while ANME-3 are connected with *Desulfobulbus* (DBB) (Niemann et al., 2006). ANME-1, as an anaerobic methanotroph ecotype, often forms monospecific chains without attached bacterial partner (Maignien et al., 2012). The ANME-2-dominated community presents significantly higher AOM rates in anoxic incubation than that of ANME-1 (Nauhaus et al., 2005).

It has been estimated that about 75% of migratory methane emitted from subsurface reservoirs is microbially consumed by ANMEs during AOM (Boetius and Wenzhöfer, 2013). In addition, AOM associated with sulfate reduction is responsible for increasing sulfide and dissolved inorganic carbon, promoting the precipitation of authigenic carbonates (Liang et al., 2017), and creating a favorable habitat for benthic megafaunal and microbial communities. For instance, the biomass of benthic communities at cold seep sites is up to tens of kilograms per square meter, exceeding by orders of magnitude the biomass in nearby seabed sediments without seeps (Zhang et al., 2002; Zhang C. L. et al., 2003; Levin, 2005). The benthic communities at cold seep sites include motile sulfide-oxidizing bacteria (Sahling et al., 2002; Zhang et al., 2005; Niemann et al., 2006), bivalve *Calyptogena* clams, and tubeworms (Cordes et al., 2005; Niemann et al., 2006). Therefore, the AOM process not only reduces methane leakage but also plays an important role in changing marine ecological habitats.

Recently, ANME communities were investigated using high-throughput sequencing technology in the sediments of the South China Sea (SCS) (Niu et al., 2017; Cui et al., 2019; Zhuang et al., 2019). However, these studies only focused on the overall microbial communities at the DNA level, without distinguishing among active, dormant, and dead microbes. DNA molecules are much more resistant to degradation and can be detected in dead cells (Lorenz and Wackernagel, 1987; Karl and Bailiff, 1989). Therefore, DNA-based abundance estimates could include both the extant and necromass of microbial communities. In contrast, high-throughput sequencing technologies focusing on RNA can be used to evaluate relative metabolic activities due to RNA relatedness to enzyme production and their sensitivity to environmental changes (Charvet et al., 2014; Pawlowski et al., 2014). Several studies have successfully determined the microbial composition in the water column of the SCS using both DNA and RNA investigations (Xu et al., 2017; Wu and Liu, 2018). However, the survey of sedimentary microbial communities conducted by employing DNA and RNA molecules is currently lacking, especially in SCS cold seep unities.

Various cold seeps have been identified on the northern continental slope of the SCS (Han et al., 2008; Feng and Chen, 2015; Liang et al., 2017; Hu et al., 2019). Among them, Formosa Ridge (site F) and Haima cold seeps are known as active seeps (Feng and Chen, 2015; Liang et al., 2017). Observations using a remotely operated vehicle (ROV) confirmed the existence of massive authigenic carbonate crusts and various benthic faunal assemblages, such as dead bivalves (*Calyptogena* sp.), living tubeworms (*Paraescarpia echinospica*), and living mussels (*Bathymodiolus plantifrons*) (Feng and Chen, 2015; Liang et al., 2017). Large numbers of ANMEs have been found in sediments from Haima cold seeps through DNA investigation, including ANME-1a, ANME-1b, ANME-2a/b, and ANME-2c (Niu et al., 2017; Guan et al., 2018; Cui et al., 2019; Zhuang et al., 2019). In 2018, two weak seeps (ROV05 and ROV07, 5.22 km apart) were newly discovered using the Haima ROV. They are located about 110 km to the northeast of the Haima cold seeps below 1,721–1,753 m of water depth (Feng et al., 2020). These two seeps are obviously different from Haima cold seeps since they belong to different stages of cold seep evolution and harbor simple megafauna (mainly including *Calyptogena*) communities. Large amounts of methane gas hydrates have been revealed in the area where the two seeps occur, with hydrate depths of 15–160 m and 8–174 m below sediment surface at sites ROV05 and ROV07, respectively (Liang et al., 2019; Wei et al., 2019; Ye et al., 2019).

This comparative study was conducted at the DNA and RNA levels in combination with network analysis to evaluate the changes in microbial (archaeal and bacterial) communities in these two new seeps, aiming to (1) determine and contrast the active microbial communities involved in AOM on the northern slope of the SCS; (2) investigate the network correlations and partnerships between ANMEs and other microbes at these differing sites; and (3) explore the variation of ANMEs in different stages of cold seep evolution.

## Materials and Methods

### Site Description and Sampling

**Figure 1:**
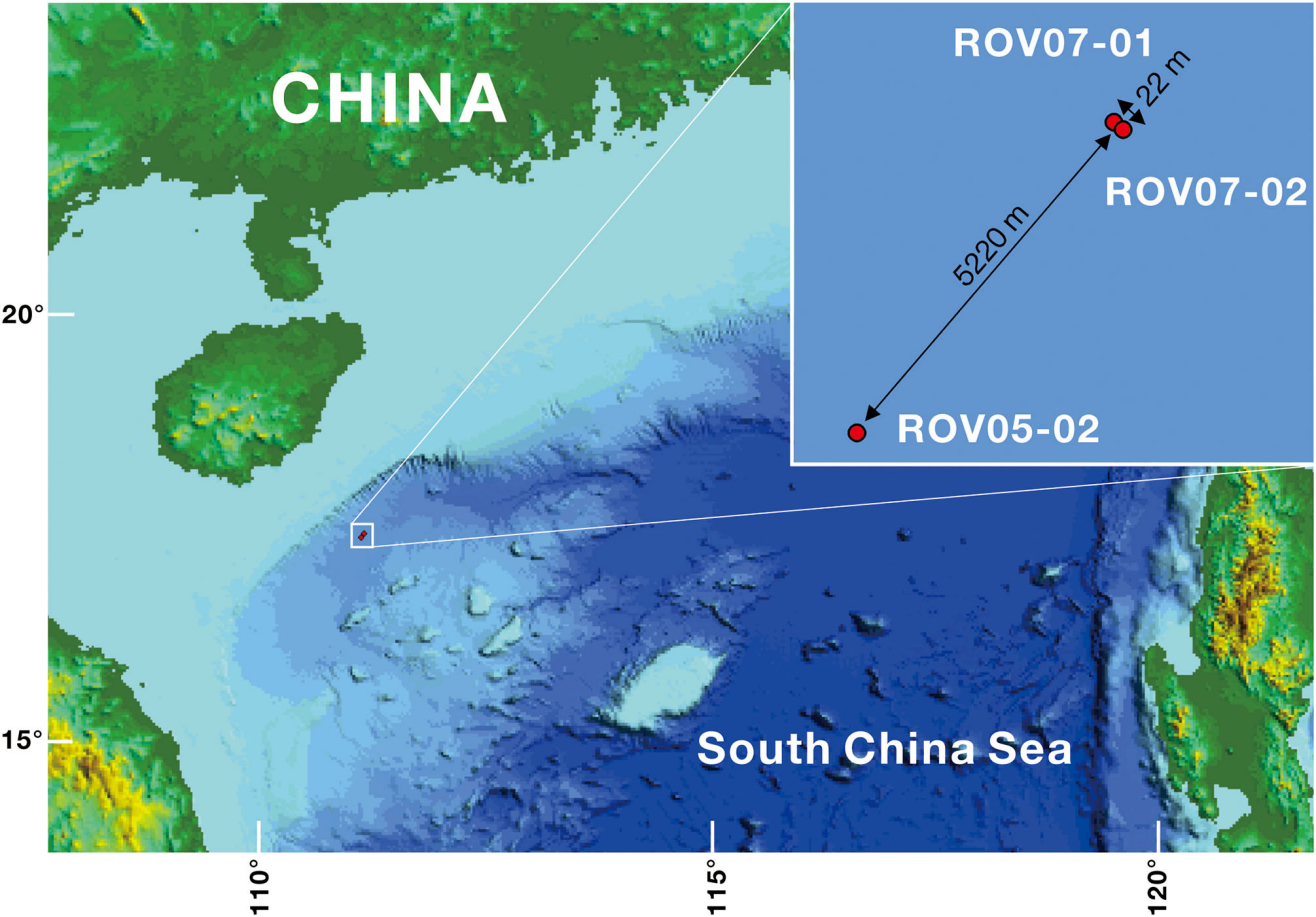
Locations of the cold seeps ROV05 and ROV07 in the northern part of the SCS. ROV05-02 samples were collected from ROV05, and ROV07-01 and ROV07-02 samples from ROV07.

Sediment samples were collected with the Haima ROV from the new weak seeps (ROV05 and ROV07) during the cruise HYLH2018-01 (Figure 1). ROV05 was observed to show a small mud mound (diameter: 10 m) colonized by dense populations of living clams (*Calyptogena* sp., Supplementary Figure 1). One push core was carried out near the clam patch and was named ROV05-02. Another surface sediment sample with living clams was collected by an ROV gripper and was named LC (living clams). ROV07 was observed to show that the shells of dead clams (*Calyptogena*) and carbonate crusts were scattered over a few 100 m. Two push cores were obtained from the sediments near the shells of dead clams and the sediments near the carbonate crusts and were named ROV07-01 and ROV07-02, respectively. In addition, another sediment sample was collected in dead clam shells near the push core ROV07-01 by an ROV gripper and was named CS (Clam shells). In brief, the site of ROV05-02 was colonized by living *Calyptogena* clams with 1,721 m of water depth (Supplementary Figure 1), the site of ROV07-01 accumulated deceased *Calyptogena* clam shells with 1,753 m of water depth, and the site of ROV07-02 was located near carbonate crusts with few clam shell debris (1,751 m of water depth). The site of ROV05-02 is far away from ROV07-01 (5.22 km apart), while site ROV07-01 is 20 m away from ROV07-02.

Each of the sediment cores was cut into halves on board, whereby one half was subsampled for molecular analysis and the other half was collected for geochemical analysis of pore water. All sediment samples were collected aseptically from 5cm-thick layers using sterile spoons, and were then frozen at −20°C until further processing. The dissolved inorganic carbon (DIC), δ^13^C_DIC_ values, total alkalinity (TA), and cation-anion concentrations (${\text{SO}}_{4}^{2-}$, ${\text{NO}}_{3}^{-}$, Cl^−^, Mg^2+^, Ca^2+^, K^+^, and Na^+^) were determined as previously described (Feng et al., 2020). Particle size was measured using a Malvern Mastersizer2000 (Malvern Panalytical, UK).

### DNA and RNA Extraction and PCR and Quantitative PCR Amplification

The total DNA and RNA of the sediments were extracted and purified using the E.Z.N.A.® soil DNA Kit and the E.Z.N.A.® soil RNA Mini Kit (Omega Bio-tek, Norcross, GA, U.S.), respectively. Aliquots of rRNA were reversely transcribed using HiScript® Q RT SuperMix (Vazyme Biotech Co., Ltd.) according to the manufacturer's protocols after purification. DNA concentration and quality were determined using a NanoDrop 2000 UV-vis spectrophotometer (Thermo Scientific, Wilmington, USA) and 1% agarose gel electrophoresis, respectively.

The DNA amplification were performed by a thermocycler PCR system (GeneAmp 9700, ABI, USA) through the following process: 5 min of denaturation at 95°C; 35 cycles of sub-process constituting 30 s at 95°C, 30 s for annealing at appropriate temperature according to targeting genes (listed in Supplementary Table 1), and 1 min for elongation at 72°C successively; a final extension at 72°C for 10 min. PCR reactions were performed in triplicate 20 μL mixture containing 10 μL of 2X Taq Plus Master Mix, 0.8 μL of each primer (5 μM), 7.4 μL of ddH_2_O, and 1 μL of template DNA. Primer-pairs were used to amplify the different microbial communities as shown in Supplementary Table 1.

Quantitative PCR (qPCR) was performed using an ABI7500 Real-Time system (Applied Biosystem, U.S.A.). The qPCR reaction was performed according to the following process: 5 min of denaturation at 95°C, followed by 40 cycles of sub-process consisting of 5 s at 95°C, 30 s for annealing at appropriate temperature according to targeting genes (listed in Supplementary Table 1), and 40 s for elongation at 72°C. The qPCR reactions were performed in triplicate 20 μL mixture containing 10 μL of ChamQ SYBR Color qPCR Master Mix, 0.8 μL of each primer (5 μM), and 1 μL of template DNA. Standard templates were generated using a dilution series of purified plasmids with *R*^2^ values greater than 0.99.

### Illumina MiSeq Sequencing

16S rDNA and rRNA sequencing of target microbial (archaeal and bacterial, Supplementary Table 1) communities were conducted with an Illumina MiSeq platform (Illumina, San Diego, USA) according to the standard protocols of Majorbio Bio-Pharm Technology Co. Ltd. (Shanghai, China). Low-quality sequences were demultiplexed, filtered by Trimmomatic, and merged by FLASH (Bolger et al., 2014). After removing chimeric sequences using UCHIME (Edgar et al., 2011), high-quality sequences were clustered into operational taxonomic units (OTUs) at similarity levels of 97% using UPARSE (version 7.1, http://drive5.com/uparse/). The taxonomy was assigned to representative sequences by RDP Classifier algorithm (http://rdp.cme.msu.edu/) against the Silva (SSU132) 16S rRNA database using a confidence threshold of 70% (Quast et al., 2013).

### Statistical Analysis

The differences of archaeal and bacterial structure between DNA and RNA sequences were analyzed in R language using Bray Curtis method. Principal Component Analysis (PCA) was performed to evaluate the variations at different sites based on RNA level. Archaeal sequences were aligned with BLAST hits from GenBank using the MEGA software (Hall, 2013), followed by manual adjustments. Phylogenic tree was generated based on maximum likelihood analysis. The robustness of inferred topology was measured by bootstrap resampling (1,000). The tree was then drawn in the web-based interactive tree of life (iTOL) (Letunic and Bork, 2016).

The co-occurrence network was analyzed in R language (V.3.2.4) and visualized by using Cytoscape (Shannon et al., 2003). The archaeal OTUs (the top 50, representing 87.2–94.6% of total archaeal reads) and bacterial OTUs with the highest absolute abundance (the top 200, representing 61.8–74.2% of total bacterial reads) were firstly selected to be analyzed based on Pearson correlation matrix in R language. The absolute abundance was based on their population and equal to their relative abundance in DNA sequence libraries multiplied by archaeal and bacterial DNA quantification, respectively. The significant correlations (coefficient >0.9 or < −0.9, *P* ≤ 0.01) between two OTUs were secondarily selected and visualized using Cytoscape.

Redundancy analysis (RDA) was performed using PRIMER V6.1.16 & PERMANOVA+ V1.0.6. The distance matrix of archaeal communities serves as response variables and was calculated by the method of Bray Curtis similarity. Meanwhile, predictor variables of environmental factors were selected by the step-wise method through *R*^2^ criterion and further tested by Monte-Carlo significance test through 999 permutations.

## Results

### Geochemical Characterization

**Figure 2:**
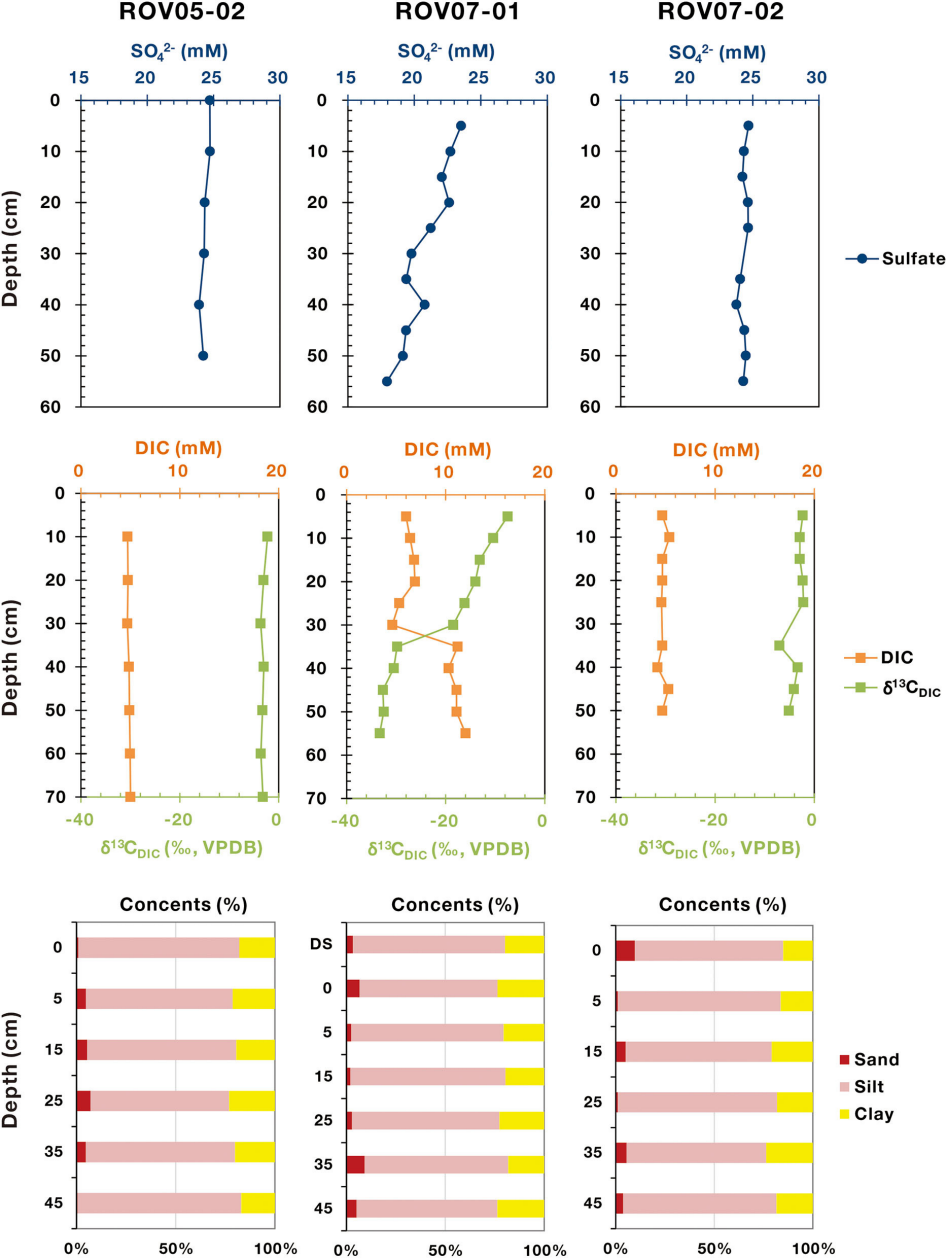
Geochemical profiles of sulfate, DIC, δ^13^C_DIC_, and particle sizes in the sediments of cold seeps. The sulfate, DIC, and δ^13^C_DIC_ data are quoted from Feng et al. (2020).

Comparing and contrasting data revealed similarities and contrasts between the three sample locations (Figure 2). Seawater impacts on shallow sediments were common across sites with sulfate ~25 mM, DIC ~5 mM and δ^13^C_DIC_ ~ −20‰. Overall, it appeared that seawater impacts dominated in 0–20 cm depths. Methane concentration was likely undervalued due to outgassing during sampling, however, the methane range of the push cores clearly differed. Site ROV07-02 exhibited very low methane concentrations (0.19–0.54 μM) throughout the 60 cm profile as well as DIC and ${\text{SO}}_{4}^{2-}$ similar to overlying waters. Below ~30 cm there was a slight increase in DIC with concomitant decrease in δ^13^C_DIC_. Meanwhile, the sulfate concentrations remained constant at 24–25 mM in the sediments of ROV07-02 indicative of insignificant sulfate reduction. Combined with the lack of sulfide smell, these findings suggest low biological activity in the 60 cm core ROV07-02. Core ROV05-02 appeared similar to ROV07-02 except for higher methane concentrations (0.21–3.71 μM). These results suggested a significant methane energy source available in the sediments of ROV05-02 and perhaps some methane production or at least accumulation, suggestive of biological activity.

In stark contrast were sediments from site ROV07-01. The sulfate concentrations decreased rapidly from 23.52 to 17.95 mM within 55 cm depth. Unlike sediments from ROV07-02 and ROV05-02, the ROV07-01 sediments exhibited a strong smell of sulfide, indicative of a highly reducing environment. Concomitantly, methane was presented at a higher level in the ROV07-01 sediments rising from 0.49 μM to 7.03 μM. Further evidence of enhanced microbial activities in ROV07-01 sediments was increased DIC at depth. The DIC content was ~5 mM at a depth of 0–30 cm. Then it greatly increased to 11.2 mM at a depth of 35 cm, and finally remained constant again at a depth of 35–55 cm. While DIC at the sediment water interface was likely in equilibrium with the overlying waters, the DIC at 60 cm depth was 2 times higher than that of the overlying waters. In parallel, the δ^13^C value of the DIC in the sediments of ROV07-01 presented an opposite trend. It was slightly negative at a depth of 0–30 cm (−7.5~-18.5‰), similar to that of ROV05-02 and ROV07-02, then became largely depleted at a depth of 35 cm, and finally remained constant (~-32‰) at a depth of 35–55 cm. The depletion of δ^13^C_DIC_ is characteristic of biological oxidation of reduced carbon. Presence of sulfide, removal of sulfate in the upper 5+ cm depths, and increased DIC with concomitant decreasing δ^13^C_DIC_ provided multiple lines of evidence for significant anaerobic activities and likely extending below the 60 cm core in ROV07-01 sediments.

Profiles of particle size of the three sediment cores were similar, with a high abundance of silt content ranging from 69.7 to 82.6%. As for the sand content (0.5–9.6%), it showed a high abundance in middle layers in core ROV05-02 (up to 7.0%), with the highest abundance occurring at 35 cm depth in core ROV07-01.

### Quantification of Archaea, Bacteria and ANME Subgroups

**Figure 3:**
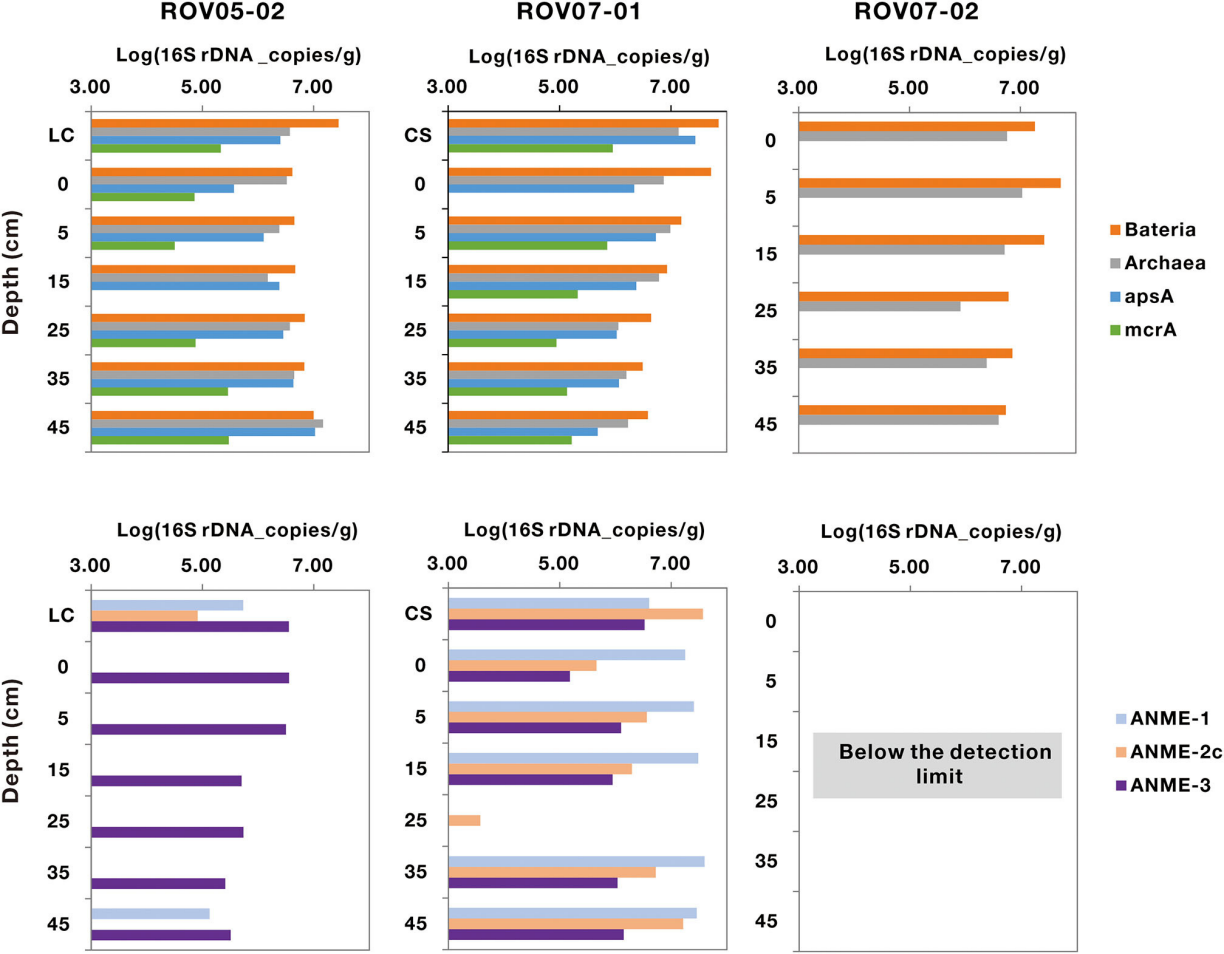
Quantification of the populations of archaeal, bacterial, *apsA, mcrA*, ANME-1, ANME-2c, and ANME-3 genes in cold seep sediments. LC and CS represent the sediment samples with living clams and die-off clam shells at surface sediment, respectively. The function gene *apsA* was not measured in core ROV07-02; the genes of *mcrA*, ANME-1, ANME-2c, and ANME-3 were below the detection limit.

Bacteria were nearly three times more abundant than archaea in the three sites, with abundance ranging from 1.71 × 10^7^ to 5.24 × 10^6^ copies/g (wet weight), respectively (Figure 3). Sediments of ROV05-02 and ROV07-01 exhibited significant abundance of the *apsA* functional gene ranging from 3.84 × 10^6^ to 5.74 × 10^6^ copies/g wet weight sediments on average, with the presumably active ROV07-01 sediments evidencing 1.65 times more than ROV05-02 sediments. The functional gene *mcrA* for methane metabolism was retrieved from ROV05-02 and ROV07-01, with abundance ranging from 1.64 × 10^5^ to 3.71 × 10^5^ copies/g (wet weight), respectively. Again, the higher abundances were noted in the more active and diverse ROV07-01 samples. Unsurprisingly, *mcrA* gene was not detected in the presumably lower activity sediments of ROV07-02.

The abundance of ANME-1, ANME-2c, and ANME-3 could only be quantified in the sediments of ROV05-02 and ROV07-01 by Q-PCR. All ANME groups were below detectable limits in each depth in the lower activity ROV07-02 core. ANME-1 and ANME-2c genes were more abundant in the sediments of ROV07-01, with an average of 2.44 × 10^7^ and 9.25 × 10^6^ copies/g wet weight sediments, respectively. However, they were hardly detected from most of the sediments of ROV05-02. ANME-3 gene was detectable from the sediments of ROV05-02 and ROV07-01, averaging 1.71 × 10^6^ and 1.35 × 10^6^ copies/g wet weight sediments, respectively.

### Phylogenetic Diversity of Metabolically Active Archaea

The differences in archaeal and bacterial community structures were shown between DNA and RNA sequence libraries. Significant differences in archaeal structural patterns were shown in sediment cores ROV07-01 (*R* = 0.68, Sig. = 0.003) and ROV07-02 (*R* = 0.63, Sig. = 0.003) (Supplementary Table 2). Significant differences in bacterial structural pattern were observed in the sediment cores ROV05-02 (*R* = 0.50, Sig. = 0.003) and ROV07-01 (*R* = 0.72, Sig. =0.001; bacterial RNA communities were not detected). In general, differences between DNA and RNA sequence libraries of the sediment core ROV07-01 were greater than that of the sediment core ROV05-02.

**Figure 4:**
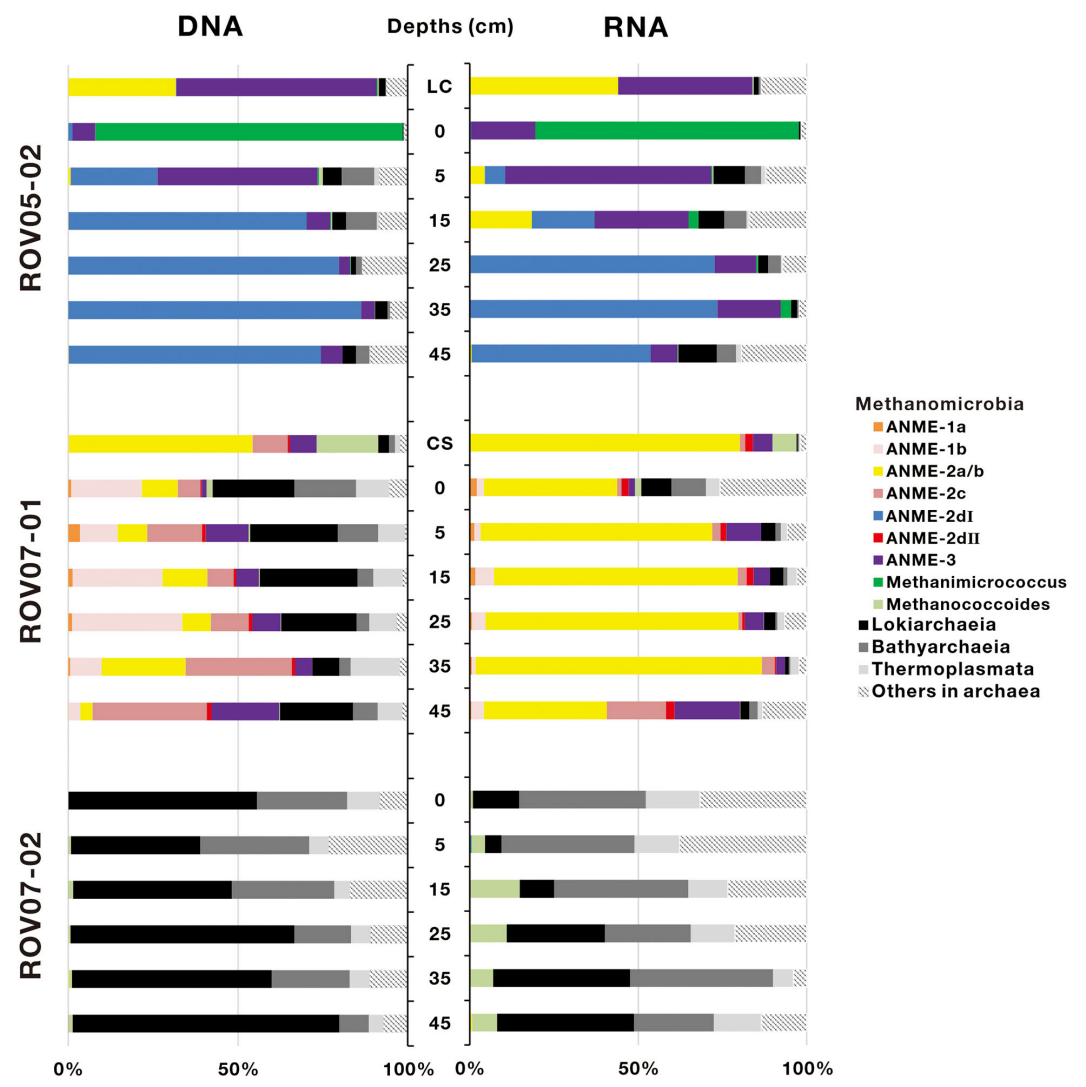
Archaeal community composition based on 16S rDNA and 16S rRNA-derived sequences in cold seep sediments. LC and CS represent the sediment samples with living clams and die-off clam shells at surface sediment, respectively.

A total of 367,333 16S rRNA sequences of archaea were obtained from 20 sediment samples, and were assigned to 919 archaeal OTUs (Supplementary Table 3). In general, both DNA and RNA sequences retrieved were mostly affiliated with methane-metabolizing archaea (ANMEs and methanogens, Figure 4), with the exception of sediments of ROV07-02, which consistently appeared low in both methane-metabolizing biomass and likely activity. Site ROV05-02 exhibited considerable abundance of ANMEs by DNA sequencing and likely activity as exhibited by RNA sequencing. In addition, the live clam area (ROV05-02) at the surface sediment exhibited significant ANME-3 and methanogenic DNA and RNA, at depth ANME-2d were in higher preponderance than other archaea. Site ROV07-01 revealed a diverse community of methanogens and ANMEs with near corresponding proportions of RNA suggesting diverse activity as well.

ANME-2d increased proportionally with depth and became predominant in deep sediment layers in both DNA and RNA sequence libraries at the ROV05-02 site. In contrast, ANME-2d accounted for a small proportion of archaea at the ROV07-01 site and was not observed at ROV07-02. ANME-2d was further divided into two clades, namely ANME-2dI and ANME-2dII, with a similarity of 83.88–87.91% (Supplementary Figure 2). The two clades were respectively derived from the sediments of ROV05-02 and ROV07-01. The RNA sequences of ANME-2a/b were significantly predominant in all sediment layers (averaging 65.4% of archaea) at ROV07-01. The DNA and RNA sequences of ANME-2a/b were detected in shallow sediment layers but were present at low percentage in archaea at the site of ROV05-02. Interestingly, ANME-2a/b was dominant in sediments with living *Calyptogen*a clam or dead clam shells. ANME-3 was dominant in shallow sediment layers at site ROV05-02. In contrast, it showed a low relative abundance of archaea in both DNA and RNA sequence libraries at site ROV07-01. The sequences affiliated to ANME-1a, ANME-1b, and ANME-2c were only obtained from the sediments of ROV07-01. Methanogen sequences belonging to *Methanimicrococcus* were only predominant in the surface sediment layer at site ROV05-02, and *Methanococcoides* occurred predominantly in the sediments with dead clam shells. In addition, *Methanococcoides* were dominant in all sediment layers at site ROV07-02 in RNA libraries, although few ANME communities were detected at this site. The amplification of *Lokiarchaeia, Bathyarchaeia*, and *Thermoplasmata* also occurred as major groups at site ROV07-02.

PCA analysis was performed to examine variations of the active archaeal communities at the RNA levels across the three different cores, and two optimal PCA dimensions (37.1% by first axis and 22.1% by second axis) were presented in Supplementary Figure 3. The results confirmed that archaeal communities in most samples collected from each push core were clustered together, substantiating the different communities and activities in each site. In detail, ANME-2dI and *Methanimicrococcus* showed a preference for the sediments of ROV05-02; ANME-2a/b, ANME-2dII, ANME-1a, and ANME-1b were mainly detected in the sediments of ROV07-01; *Methanococcoides, Lokiarchaeia, Bathyarchaeia*, and *Thermoplasmata* were mostly located in the sediments of ROV07-02. The lack of ANMEs in ROV07-02 was in contrast to the diverse and apparently active ANMEs in ROV07-01.

### Phylogenetic Diversity of Metabolically Active Bacteria

**Figure 5:**
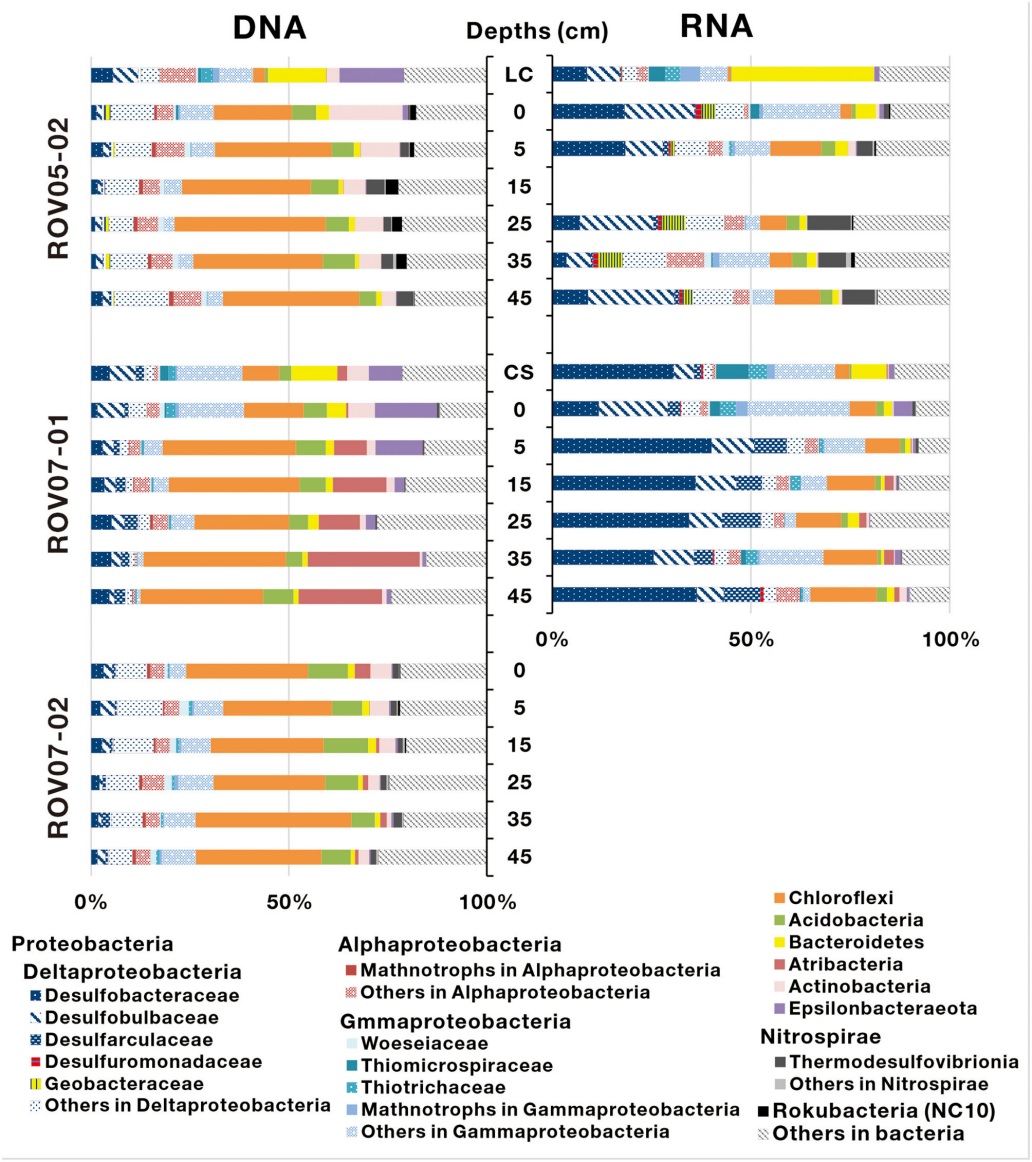
Bacterial community composition determined based on 16S rDNA and 16S rRNA-derived sequences in the sediments of cold seeps. LC and CS indicate the sediment samples with living clams and die-off clam shells at surface sediment, respectively.

A total of 674,266 16S rRNA sequences of bacteria were obtained from 16 sediment samples (Site ROV05-02 and ROV07-01), and were assigned to 7,083 bacterial OTUs (Supplementary Table 3). The bacterial phyla mainly consisted of *Proteobacteria, Chloroflexi, Acidobacteria, Bacteroidetes, Atribacteria, Actinobacteria*, and *Epsilonbacteraeota* (Figure 5). Among them, *Proteobacteria* were predominant in both DNA and RNA sequence libraries (especially in RNA sequence libraries) at the site of ROV07-01 (averaging 69.8%). According to RNA sequence libraries of the sediments of ROV05-02 and ROV07-01, *Deltaproteobacteria* was the most important group of Proteobacteria. Most of the sequences belonging to *Deltaproteobacteria* were affiliated with sulfate-reducing bacteria from families of *Desulfobacteraceae, Desulfobulbaceae, Desulfarculaceae*, and *Desulfuromonadaceae*. More abundant sulfate-reducing bacteria in ROV07-01 even further substantiated the likely highly anaerobic conditions in ROV07-01 sediments and likely low redox condition in ROV05-02 sediments. Aerobic methanotrophs of *Gammaproteobacteria* that are related to type I methanotrophs occurred in RNA sequences and showed more abundance in shallow sediment layers. However, aerobic methanotrophs of *Alphaproteobacteria* related to Type II methanotrophs occurred in most sediment samples in DNA sequence libraries and were barely detected in RNA sequence libraries. Giant sulfide-oxidizing bacteria of *Gammaproteobacteria* represented by *Beggiatoa* showed relatively higher abundance at the site of ROV05-02, especially in the surface layer. However, sulfide-oxidizing bacteria of *Ectothiorhodospiraceae* and *Chromatiaceae* within *Gammaproteobacteria* showed a high abundance in the sediments of ROV07-01. *Geobacteraceae* consisting almost entirely of *Geothermobacter* were abundant in the sediments of ROV05-02; however, they were rarely detected at the sites of other push cores. *Thermodesulfovibrionia* affiliated to *Nitrospirae* was also found from the sediments of ROV05-02. Sequences that belong to *Chloroflexi* were dominant throughout all sediment cores in DNA sequence libraries; other sequences affiliated to *Acidobacteria, Bacteroidetes, Atribacteria*, and *Actinobacteria* were found in a low proportion in all sediments. Active bacteria were not ascertained at site ROV07-02 due to their low abundance.

### Network Co-occurrence Analysis Regarding Archaeal Anaerobic Methanotrophs

**Figure 6:**
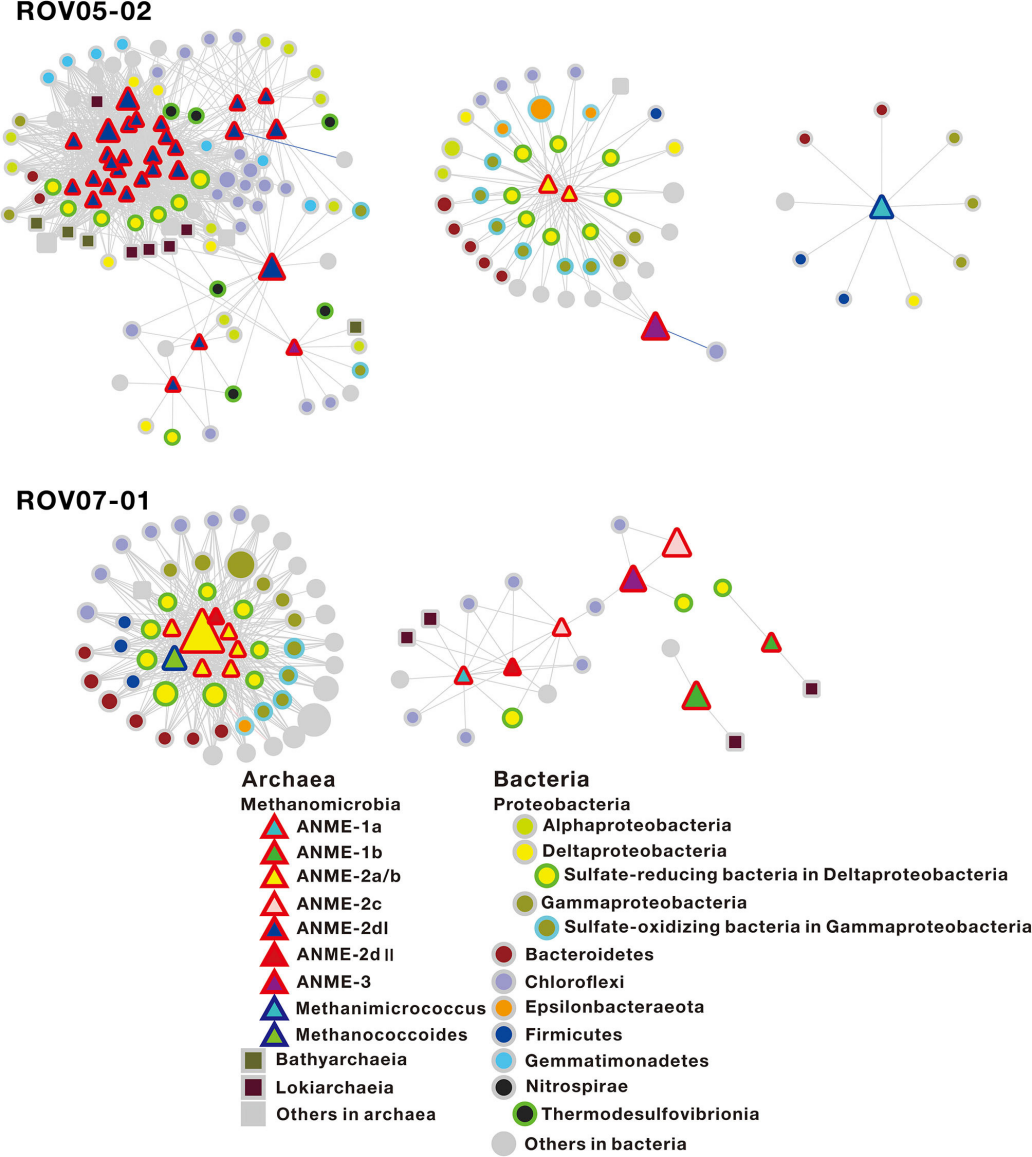
Sub-network co-occurrence patterns regarding the relationship of the methane-metabolizing archaea with other microbial taxa in the sediments of ROV05-02 and ROV07-01 in the cold seeps. The sub-network of the sediments of ROV07-02 is not presented, as only one node belongs to methane-metabolizing archaea. The red-sided triangles, blue-sided triangles, squares, and circles in the network represent ANMEs, archaeal methanotrophs, other archaea, and bacteria, respectively. Node size indicates the relative abundance of OTUs. Edges represent co-occurrence relationships between nodes (coefficient >0.9 or < −0.9, *P* ≤ 0.01). Gray edges and blue edges represent positive and negative relationships, respectively. The specific OTUs correlated with ANME-2dI and ANME-2a/b are shown in Supplementary Table 5.

To gain insights into potential biotic interplays, where ANMEs exhibited their distinct metabolic capabilities within communities and strong correlations with their syntrophic partnerships, network co-occurrence patterns of archaeal and bacterial populations were determined based on Pearson correlation analysis (Supplementary Figure 4). In order to focus on methane-metabolizing archaea, the sub-networks regarding relationships between the methane-metabolizing archaea with other microbial taxa were selected (Figure 6).

At the site of ROV05-02, ANME-2a/b (2 nodes), ANME-2dI (28 nodes), ANME-3 (3 nodes), and *Methanimicrococcus* (1 node) were represented in the co-occurrence network, with ANME-2a/b and ANME-2d being present in the centers of different modules. In general, ANME-2dI tended to be connected with sulfate-reducing bacteria, such as *Desulfatiglans* (*Desulfarculaceae*), Sva0081 sediment group (*Desulfobacteraceae*), unclassified groups (*Desulfobulbaceae*), and *Thermodesulfovibrionia* (*Nitrospirae*) (Supplementary Table 5). In addition, ANME-2dI most likely had indirect trophic relationships with other microorganisms, including *Dehalococcoidia* (*Chloroflexi*), JG30-KF-CM66 (*Chloroflexi*), *Gemmatimonadetes, Bathyarchaeia*, and *Lokiarchaeia*. ANME-2a/b tended to be connected with multiple sulfate-reducing bacteria and sulfide-oxidizing bacteria, with the former including SEEP-SRB1 (*Desulfobulbaceae*), SEEP-SRB4 (*Desulfobulbaceae*), *Desulfocapsa* (*Desulfobulbaceae*), and the latter including *Gammaproteobacteria* and *Epsilonbacteraeota* (such as *Woeseiaceae, Thiomicrospiraceae, Thiotrichaceae, Sulfurovum*, and *Sulfurimonas)* (Figure 6). In addition, ANME-3 was present on the margin of ANME-2a/b and ANME-2d modules or isolated alone. The network analyses were insightful in delineating distinct relationships between consortia such as ANME-2dI/Deltaproteobacteria. These were distinct from the ANME-2a/b/Deltaproteobacteria and ANME-2a/b/*Gammaproteobacteria* networks. At site ROV07-01, ANME-1a (1 node), ANME-1b (2 nodes), ANME-2a/b (6 nodes), ANME-2c (2 nodes), ANME-2dII (2 nodes), ANME-3 (1 node), and *Methanococcoides* (1 node) were represented in the co-occurrence network. ANME-2a/b was still in the center of the subnetwork module (Figure 6), where the connected taxa were similar to those at site ROV05-02. In addition, two nodes belonging to ANME-2dII and *Methanococcoides* were also present in the module center. For site ROV07-02, the sub-network was not presented since nodes belonging to methanogens and ANMEs were lacking (Supplementary Figure 4). *Chloroflexi* were connected with other microbial nodes. In Archaea, *Bathyarchaeia* preferred connections with other groups; however, *Lokiarchaeia* preferred connections with themselves.

### The Control of Environmental Geochemical Factors on Archaeal Community Distribution

**Figure 7:**
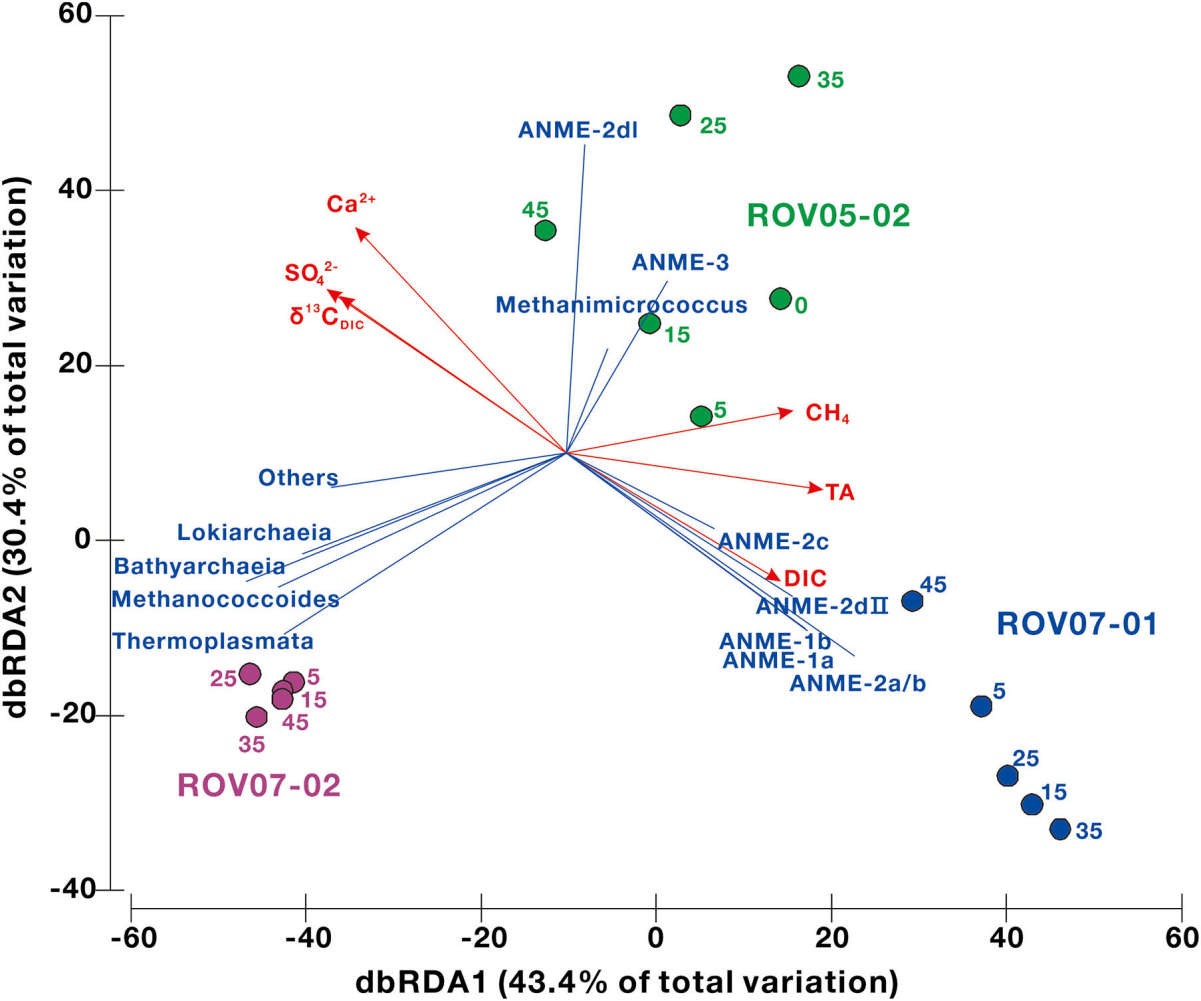
The relationships between the archaeal community structure and the environmental parameters in cold seep sediments determined using the redundancy analysis (RDA). Only significant factors are shown by red vectors. The number next to each node represents sample depth. The proportional contributions of different environmental factors are shown in Supplementary Table 4.

The environmental geochemical factors shaping the distribution of archaeal communities at the RNA level were analyzed by redundancy analysis (RDA) to gain a better understanding of the ecological niche of methane-metabolizing archaea. The predictor variables of methane, sulfate, DIC and δ^13^C_DIC_, TA, particle size, and Ca^2+^, Mg^2+^, Na^+^, K^+^, and Cl^−^ were selected for RDA (Figure 7, Supplementary Table 4). The results indicated that methane, sulfate, DIC and δ^13^C_DIC_, TA, particle size, Ca^2+^ were significant factors, which explained 73.7% (43.4% by first axis and 30.4% by second axis) of the variation in archaeal community distribution. Site ROV07-01 was associated with ANME-1 and−2, while site ROV05-02 was with ANME-2I and−3. For site ROV07-01 the loss of sulfate and diminishing ^13^C_DIC_ were obvious explanations of the variables in concert with the ANMEs. Methane and TA were predominantly associated with the site ROV05-02 and ROV07-01 in agreement with previous data presented that the site ROV07-02 showed little and sparse evidence of reducing conditions, methanogenesis, or methane oxidation.

## Discussion

Multiple studies have shown that ANMEs are dominant in cold seeps in the SCS (Niu et al., 2017; Cui et al., 2019; Zhuang et al., 2019). However, previous results were based on the DNA level, and thus inactive microorganisms may have been included (Luna et al., 2002; Ostle et al., 2003). The application of the experimental method at the RNA level can more accurately and authentically reflect the changes in the microbial community structure (Ostle et al., 2003; Allison et al., 2010). In this study, the structural patterns of both archaeal and bacterial communities differed substantially between DNA and RNA libraries. In RNA libraries, a high abundance of methane-metabolizing archaea and sulfate-reducing bacteria were present in archaeal and bacterial communities.

### Active Archaeal Anaerobic Methanotrophs in Cold Seeps

Active ANMEs appeared to have different ecological preferences in different sediment cores (Supplementary Figure 3). ANME-2a/b is widely distributed in global sediments, and especially dominates in cold seep environments (Knittel et al., 2005; Biddle et al., 2012). A large amount of ANME-2a/b has been detected in cold seeps in the SCS (Niu et al., 2017; Cui et al., 2019; Zhuang et al., 2019). ANME-2a/b was found throughout the entire sediment core ROV07-01 in this study. A large number of dead *Calyptogena* clam shells and carbonate precipitates accumulated in the ROV07 region (Supplementary Figure 1), suggesting that the cold seep system has developed for quite a long time. The chemical parameters exhibited sulfate removal, DIC increase and δ^13^C_DIC_ distinction with depth in site ROV07-01 (Figure 2), suggesting this site was highly developed, active and strictly anaerobic.

A large reduction or cessation of seepage activities may have occurred at one time in Haima cold seeps as reported (Liang et al., 2017), causing the covering of *Calyptogena* clam deaths. Therefore, benthic organisms did not include Bathymodiolin mussels and tubeworms, which are representative of later stage cold seeps evolution (Bowden et al., 2013). Presently, methane continues to seep, supporting archaeal anaerobic methanotrophs in ROV07-01 sediments. Compared to DNA sequences, ANME-2a/b was more predominant and the abundance of ANME-1a and ANME-1b was relatively lower at the RNA level (Figure 4).

The obvious changes in archaea community structure detected by these two methods may be related to the change in methane leakage flux in the ROV07 region. Since ANME-2 had a preference for sediments with high methane flux and showed significantly higher AOM rates while ANME-1 exhibited an opposite trend, it is speculated that an increase in methane flux may have occurred in recent times. Moreover, the abundance of sulfate-reducing bacteria significantly increased at the RNA level, suggesting that sulfate-dependent methane oxidation occurred in the sediments of ROV07-01. The abundant sulfide-oxidizing bacteria of *Ectothiorhodospiraceae* and *Chromatiaceae* (Meyer et al., 2007) within *Gammaproteobacteria* suggested the sufficient source of sulfide at this site. In addition, dense populations of ANME-2a/b discovered in the sediments with living *Calyptogena* clams (LC) or die-off clam shells (CS) might be linked to seawater sulfate and substrate availability to *Calyptogena* clams.

All ANME-2d in the samples were of mud volcano and hydrothermal origin (Pachiadaki et al., 2011; Schauer et al., 2011; Chang et al., 2012; Li et al., 2013), and were not derived from terrestrial origins (Supplementary Figure 2). The ANME-2d in the samples was further divided into sub-clades according to phylogenetic analysis, namely ANME-2dI and ANME-2dII, with an internal similarity of approximately 83.88–87.91%. The majority of ANME-2d sequences fell into ANME-2dI, which was dominant in ROV05-02 sediments (up to 73.5% in total archaea at the RNA level). ANME-2d was previously found in marine sediments such as a deep-sea gas chimney (Schrenk et al., 2003), hydrothermal vents (Schauer et al., 2011; Li et al., 2013), mud volcanos (Pachiadaki et al., 2011), and cold seep sediments (Reed et al., 2009; Vigneron et al., 2013). However, its abundance remained in extremely low proportion. Although a high abundance of ANME-2d was detected from deep submarine permafrost, the majority of sequences were derived from terrestrial areas (Winkel et al., 2018).

In this study, a high abundance of ANME-2dI was found in the sediments of ROV05-02, which might be related to the special habitat of the cold seep ROV05. It covered a single benthic fauna—living *Calyptogena* clams, which are early colonizers of short-lived (years to decades) and sulfide-rich surficial sediment patches (Bowden et al., 2013). The existence of *Calyptogena* suggested that ROV05-02 site possibly contained a small amount of sulfide, even though we did not smell it. The occurrence of giant sulfide-oxidizing bacteria of *Gammaproteobacteria* represented by *Beggiatoa* (Grünke et al., 2011) further substantiate the presence of sulfide. The chemical parameters evidencing a lack of sulfate removal, DIC increase and δ^13^C_DIC_ distinction in site ROV05-02 indicated this site was less developed and less anaerobic than ROV07-01 (Figure 2). The sediments with living *Calyptogena* (LC) contained higher proportions of Type I methanotrophs, which were often identified as symbionts in marine invertebrates, and they had higher energy efficiency over type II methanotrophs (Petersen and Dubilier, 2009). Meanwhile, the existence of living *Calyptogena* suggested that the sediments of ROV05-02 originated from the early evolutionary stage of cold seeps, which has been rarely discovered so far in nSCS.

Most of the cold seeps are hundreds or thousands of years old and are rich in diversified fauna and carbonate rocks (Vigneron et al., 2013; Feng and Chen, 2015; Liang et al., 2017). Organic matter accumulating in megafaunal tissues can be released into underlying sediments to be utilized by microbial communities (Sørensen and Glob, 1987). The high abundance of ANME-2dI in deep layers might be linked to upper *Calyptogena* clams, which could release organic matter to support ANME-2d.

ANME-3 was detected in cold seeps on the northern slope of the SCS, while previously found in the Sonora Margin cold seeps (Vigneron et al., 2013) and submarine mud volcano in the Barents Sea (Losekann et al., 2007). ANME-3 was found to be distributed in both ROV05-02 and ROV07-01 push cores and had a high abundance in shallow layers at 5 cm depth of ROV05-02 (Figure 4). It was previously reported that the ANME-3 habitat was restricted to a rather narrow horizon below surface layers affected by surface communities (Losekann et al., 2007; Vigneron et al., 2013).

*Methanococcoides* were detected in ROV07-02 sediments as unique methane-metabolizing archaea (Figure 4), or they could be involved in methane generation through reverse methanogenesis as previously suggested (Vigneron et al., 2013; Timmers et al., 2017). The relative abundance of ANMEs in the sediments of ROV07-02 significantly decreased, which was similar to that in the non-cold seep sediments on the northern slope of the SCS (Niu et al., 2017). The most abundant taxa in DNA libraries belonged to *Chloroflexi* (Figure 5). Some groups in *Chloroflexi* (such as *Anaerolineae*) are able to utilize polysaccharides and cellulose in strictly anaerobic environments (Podosokorskaya et al., 2013; Xia et al., 2016). Other sequences affiliated to *Acidobacteria, Bacteroidetes, Atribacteria*, and *Actinobacteria* that are capable of degrading organic matter (Steger et al., 2007; Hanreich et al., 2013; Nobu et al., 2016; Wegner and Liesack, 2017) were found in a low proportion in all sediments. Due to the lack of methane, the microbial communities and geochemical factors were like the background in nSCS. Considering the lack of sulfide smell or sulfate removal, little DIC or δ^13^C_DIC_ distinction, site ROV07-02 was likely poorly developed, less anaerobic, possessed less methane metabolic communities and exhibited less activity with respect to methane oxidation.

### Network Relationship Among ANMEs

Network co-occurrence analysis can be used to elucidate relationships such as syntrophic partnerships and explore the potential ecological roles of ANMEs. According to the analysis, large numbers of OTUs belonging to ANME-2d and ANME-2a/b were in the center of the sub-networks of ROV05-02 and ROV07-01 sediments, respectively (Figure 6). This suggests that ANME-2d and ANME-2a/b may interact strongly with surrounding microorganisms, directly or indirectly affecting their activity. ANME-2dII was connected with ANME-2a/b, while ANME-2dI in different modules existed separately from ANME-2a/b. Both ANME-2dI and ANME-2a/b were connected with sulfate-reducing bacteria in *Deltaproteobacteria*, suggesting that they could conduct sulfate-dependent AOM. However, ANME-2dI and ANME-2a/b were not sharing the OTUs of microbes, including the sulfate-reducing bacteria. ANME-2a/b can form syntrophic aggregates with bacterial partners, such as *Desulfosarcina* (Knittel and Boetius, 2009).

As shown by the network analysis in this study, ANME-2a/b was associated with multiple sulfate-reducing bacteria, including SEEP-SRB1 (*Desulfobulbaceae*), SEEP-SRB4 (*Desulfobulbaceae*), and *Desulfocapsa* (*Desulfobulbaceae*) (Figure 6). AOM associated with sulfate reduction performed by ANME-2a/b was also observed in incubations at high sulfate levels (Timmers et al., 2015). ANME-2a/b could be distinguished from ANME-2dI by their relationship with OTUs affiliated with *Gammaproteobateira* and *Epsilonbacteraeota* in networks. Meanwhile, most of the related OTUs belong to sulfur-oxidizing bacteria, such as *Woeseiaceae* (Marques et al., 2019), *Thiomicrospiraceae* (Eberhard et al., 1995), *Thiotrichaceae* (Fossing et al., 1995), *Sulfurovum* (Mori et al., 2018), and *Sulfurimonas* (Takai et al., 2006). This suggests that ANME-2a/b was indirectly associated with strong sulfur oxidation while coupling with sulfate reduction. In addition, ANME-2a/b was associated with a variety of other groups of microorganisms, but their specific metabolic processes require further scrutiny. Interactions between ANME-2a/b and their symbiotic bacteria might play an important role in the anaerobic environment and promote the high development of site ROV07-01.

The OTUs connected with ANME-2dI belonged to *Desulfatiglans* (*Desulfarculaceae*), Sva0081 sediment group (*Desulfobacteraceae*), and unclassified groups (*Desulfobulbaceae*) (Supplementary Table 5). ANME-2dI was also connected to the OTUs of *Thermodesulfovibrionia* (*Nitrospirae*). Some of the *Thermodesulfovibrio* were isolated from hydrothermal-related freshwater environments (Henry et al., 1994) and can perform sulfate reduction with a limited range of electron donors (Sekiguchi et al., 2008). In addition, ANME-2dI most likely had an indirect trophic relationship with other microorganisms, including *Dehalococcoidia* (*Chloroflexi*), JG30-KF-CM66 (*Chloroflexi*), *Gemmatimonadetes, Bathyarchaeia*, and *Lokiarchaeia*. Both *Dehalococcoidia* and JG30-KF-CM66 are affiliated to *Chloroflexi*. Meanwhile, the former contains the genes associated with degradation of various organic matter such as fatty acids and aromatic compounds (Wasmund et al., 2014) and the latter contains genes related to nitrite oxidoreduction (Mori et al., 2019). *Gemmatimonadetes* can be involved in polyphosphate accumulation and carotenoid production in soil (Zhang H. et al., 2003; Takaichi et al., 2010). However, their ecological function in marine sediments remains unclear. *Bathyarchaeia* is capable of utilizing recalcitrant organic matter, and it is involved in methane cycling in sediments (Biddle et al., 2006; He et al., 2016; Lazar et al., 2016). *Lokiarchaeia* are strictly anaerobic and hydrogen-dependent archaea (Sousa et al., 2016) and can degrade substances and syntrophically transfer hydrogen and electrons to bacterial partners (Spang et al., 2019; Imachi et al., 2020).

The metabolic flexibility of ANME-2d is seen in terrestrial environments, facilitating AOM coupled to multiple terminal electron acceptors (Leu et al., 2020) such as nitrate (Haroon et al., 2013), iron, or manganese oxides (Ettwig et al., 2016; Weber et al., 2017). “Ca. *Methylomirabilis* oxyfera” (NC10 bacteria) and *Geobacteraceae* have been observed to be the syntrophic bacteria of ANME-2d (Holmes et al., 2002; Arshad et al., 2015). NC10 bacteria perform denitrification coupled to methane oxidation by ANME-2d (Vaksmaa et al., 2017). In detail, the nitrite produced through AOM by ANME-2d will be continually reduced to N_2_ or ammonia by NC10 (Raghoebarsing et al., 2006; Hu et al., 2011). *Geobacteraceae* can perform iron reduction coupled to AOM (He et al., 2018). *Geothermobacter*, one genus of *Geobacteraceae*, is a representative of iron-reducing microbes that transfer electrons extracellularly to iron oxides (Kashefi et al., 2003; Li et al., 2020).

In this study, the secondary pore water sampling analysis (in May 2020) found that the sediments of ROV05-02 contained extremely low nitrate concentration but high bivalent iron concentration (up to 33.68 μmol/L, unpublished data). The higher relative abundance of *Geothermobacter* in RNA sequence libraries probably indicates that *Geothermobacter* was involved in iron reduction in the sediments of ROV05-02. The lack of correlation between ANME-2dI and *Geothermobacter* suggests that *Geothermobacter* may not be a syntrophic partner of ANME-2dI. However, the correlation analysis might have been influenced by some unknown factors. In general, the network of ANME-2dI with their relative microbes in site ROV05-02 was significantly different from that of ANME-2a/b, possibly caused by unique metabolic characteristics of ANME-2dI or the lower redox condition than site ROV07-01.

Molecular data at the DNA level highlighted the high-proportion presence of ANME-1, ANME-2c, and ANME-3 relatives in the sediments of ROV05-02 and ROV07-01. However, they were associated with bacteria at a low level. At site ROV07-02, the lack of methanogens and ANMEs associated with other OTUs indicated that the effect of methane-metabolizing archaea on the whole community structure is extremely small, further substantiating that site ROV07-02 was poorly developed and low activity with respect to methane metabolism.

### Control of Geochemical Factors Over ANME Distribution

The distribution and metabolism of ANMEs can also be linked to geochemical composition, such as methane, sulfate, and DIC (Timmers et al., 2015; Bowles et al., 2016). As shown by the RDA analysis in this study, methane, sulfate, DIC, and δ^13^C_DIC_, TA, and calcium could be the significant factors relating to the archaeal distribution at the RNA level (Figure 7). These environmental factors were dominantly associated with ANMEs in ROV07-01 sediments. Due to predominance of ANME-2a/b in RNA libraries of site ROV07-01 and its high correlation with sulfate-reducing and sulfide-oxidizing bacteria, the majority of AOM could be mediated by ANME-2a/b, utilizing sulfate and producing DIC and TA as revealed by the depleted δ^13^C_DIC_ (Whiticar, 1999). This could have led to the precipitation of authigenic carbonates and induced the decrease in calcium (Hu et al., 2019). ANME-2a/b have been observed growing in incubation experiments with high sulfate and AOM activity (Timmers et al., 2015). The decrease in sulfate with depth in our study indicated that the effective AOM was likely performed by ANME-2a/b. The DIC and δ^13^C_DIC_ profile changed sharply at a depth of 30–35 cm in ROV07-01 sediments, while the archaeal community and quantification of *mcrA* genes did not show any corresponding change. The increasing sand content at this depth could explain the fact that porosity might improve AOM efficiency (Figure 2) (Treude et al., 2005). Thus, the site of ROV07-01 was highly developed, diverse, anaerobic, and active in methane oxidation, providing an environment for ANME-2a/b occurrence.

Dense archaeal populations (such as *Lokiarchaeia, Bathyarchaeia, Thermoplasmata*) at site ROV07-02 did not show similar correlations with these significant geochemical factors. This result could also be deduced from the lack of methane, sulfate removal, DIC or δ^13^C_DIC_ distinction with depth. Site ROV07-02 could be less anaerobic and less active with respect to methanogenesis, methane oxidation and related network relationships. Dominant and active ANMEs (such as ANME-2dI and ANME-3) occurred at the ROV05-02 site, suggesting the activity of methane oxidation was present. However, the data of sulfate, DIC, and δ^13^C_DIC_ with depth was not as convincing as for the site of ROV07-01. This was probably due to the weak AOM performed by ANME-2dI and ANME-3. Meanwhile, the advective import of seawater and impacts by *Calyptogena* clams (Wallmann et al., 1997; Bertics et al., 2007; Fischer et al., 2012) could also explain the unchanging environmental factors. Findings suggest the ROV05-02 site could be anaerobic in spots and in micro-niches and likely intermediary between the highly diverse and active ROV07-01 site and the poorly developed low activity ROV07-02 site.

## Data Availability Statement

The datasets presented in this study can be found in online repositories. The names of the repository/repositories and accession number(s) can be found at: https://www.ncbi.nlm.nih.gov/, PRJNA664582.

## Author Contributions

TZ and JZ conceived the study and designed the experiments. ZC provided the samples and the picture of seafloor observations. XX determined the physicochemical parameters. TZ, XX, and SC analyzed the data and wrote the manuscript. JF, JZ, QL, TP, and CZ edited and approved the final manuscript. All authors contributed to the article and approved the submitted version.

## Conflict of Interest

The authors declare that the research was conducted in the absence of any commercial or financial relationships that could be construed as a potential conflict of interest.
