# Corrigendum: Active Anaerobic Archaeal Methanotrophs in Recently Emerged Cold Seeps of Northern South China Sea

**DOI:** 10.3389/fmicb.2021.653239

**Published:** 2021-02-11

**Authors:** Tingting Zhang, Xi Xiao, Songze Chen, Jing Zhao, Zongheng Chen, Junxi Feng, Qianyong Liang, Tommy J. Phelps, Chuanlun Zhang

**Affiliations:** ^1^Guangzhou Marine Geological Survey, China Geological Survey, Guangzhou, China; ^2^Gas Hydrate Engineering Technology Center, China Geological Survey, Guangzhou, China; ^3^Southern Marine Science and Engineering Guangdong Laboratory (Guangzhou), Guangzhou, China; ^4^Department of Ocean Science and Engineering, Southern University of Science and Technology, Shenzhen, China; ^5^Shenzhen Key Laboratory of Marine Archaea Geo-Omics, Southern University of Science and Technology, Shenzhen, China; ^6^Earth and Planetary Sciences, University of Tennessee, Knoxville, Knoxville, TN, United States

**Keywords:** anaerobic methanotrophs, methane, anaerobic oxidation of methane, cold seeps, calyptogena

In the published article, there was an error in affiliation 3. Instead of “Southern Marine Science and Engineering Guangdong Laboratory, Guangzhou, China,” it should be “Southern Marine Science and Engineering Guangdong Laboratory (Guangzhou), Guangzhou, China.”

The authors apologize for this error and state that this does not change the scientific conclusions of the article in any way. The original article has been updated.

