# Supplementary material for: Active Anaerobic Archaeal Methanotrophs in Recently Emerged Cold Seeps of Northern South China Sea
Source: Front Microbiol. 2020 Dec 16;11:612135. doi: 10.3389/fmicb.2020.612135 (PMC7772427; doi:10.3389/fmicb.2020.612135)
Supplement: Supplementary file 1 [file Data_Sheet_1.docx]

Supplementary Material

# Supplementary Figures and Tables

## Supplementary Figures

**
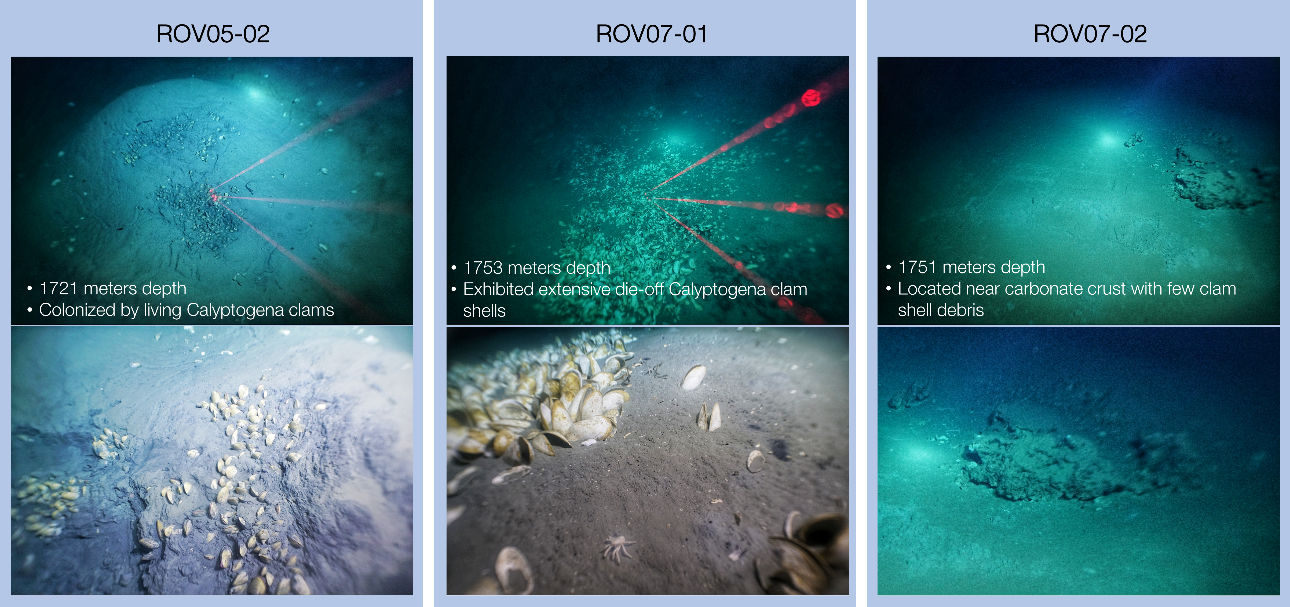
**

**Supplementary Figure S1.** Seafloor observations of sampling area at sites of ROV05-02, ROV07-01, and ROV07-02 in cold seeps.

**
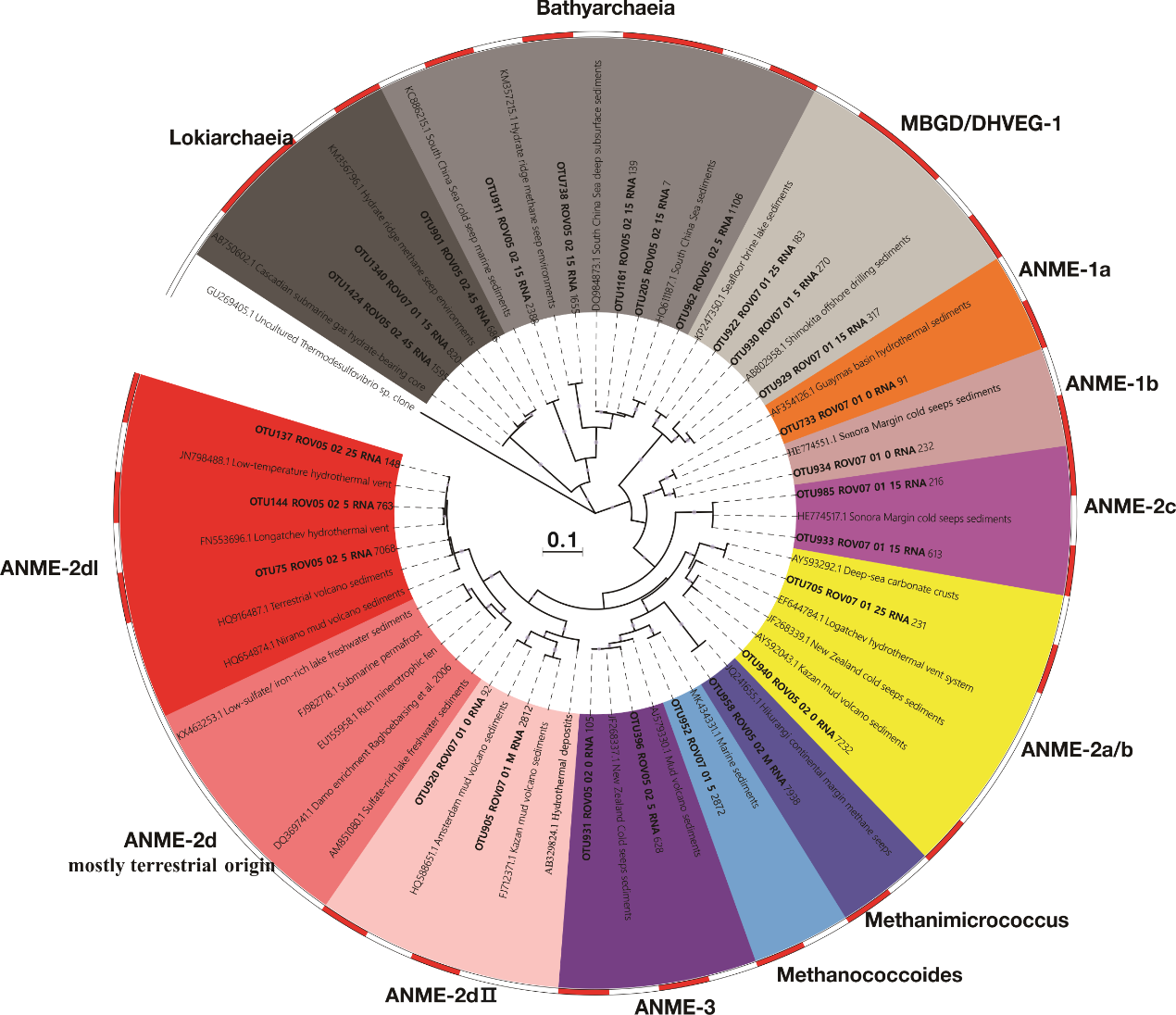
**

**Supplementary Figure S2.** Maximum likelihood phylogenetic tree of the archaeal 16S rDNA and rRNA-derived sequences for ANME and methanogen clusters. The sequences from cold seep sediments are highlighted in bold and indicated by outer red-colored arcs.

**
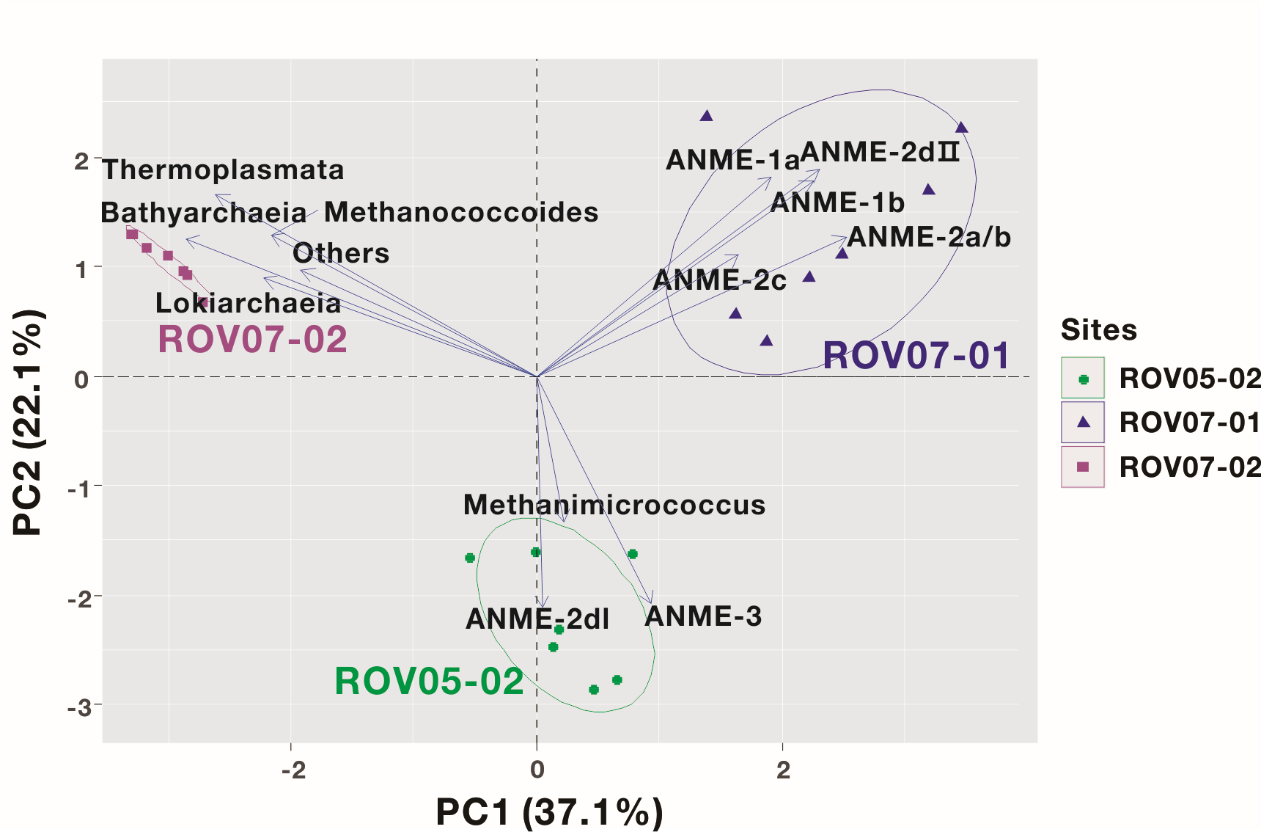
**

**Supplementary Figure S3.** PCA plots of archaeal community structure based on 16S rRNA-derived sequences in cold seep sediments.


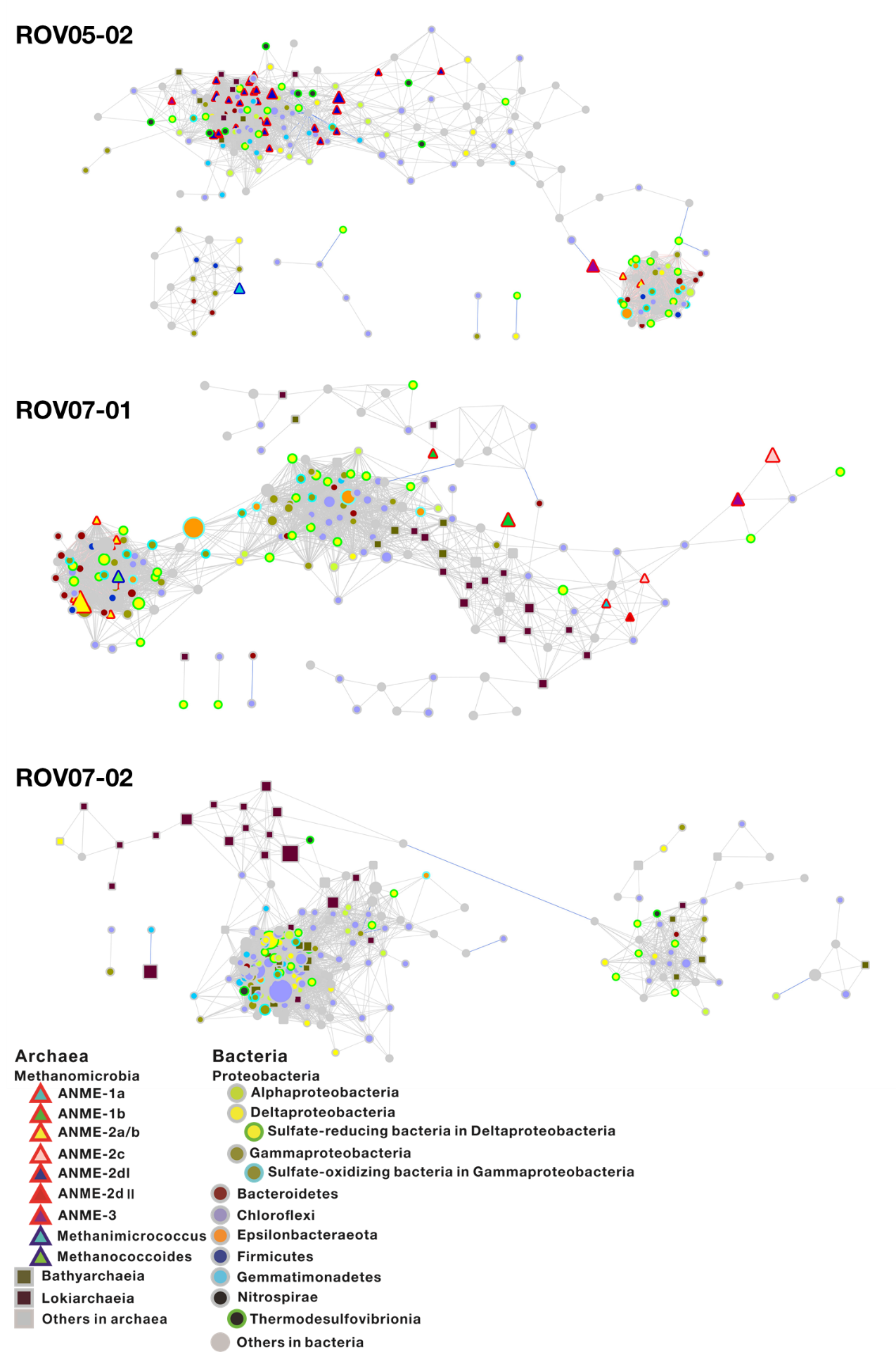


**Supplementary Figure S4.** **Network co-occurrence patterns of the archaeal and bacterial population.** The red-sided triangles, blue-sided triangles, squares, and circles in the network represent ANMEs, archaeal methanotrophs, other archaea, and bacteria, respectively. Node size indicates the relative abundance of OTUs. Edges represent co-occurrence relationships between nodes (coefficient > 0.9 or < −0.9, P value ≤ 0.01). Grey edges and blue edges represent positive and negative relationships, respectively.

## Supplementary Tables

**Supplementary Table S1.** primers used for quantitative real-time PCR and sequencing of 16S rRNA genes.

|  | Target genes | Primer names | 5’-3’ | Annealing temperature (℃) | Ref. |
| --- | --- | --- | --- | --- | --- |
| Q-PCR | Bacteria | Bac_331F | TCCTACGGGAGGCAGCAGT | 60 | (Nadkarni et al., 2002) |
|  |  | Bac_797R | GGACTACCAGGGTCTAATCCTGTT |  | (Nadkarni et al., 2002) |
|  | Archaea | Ar_787F | ATTAGATACCCSBGTAGTCC | 58 | (Yu et al., 2005) |
|  |  | Ar_915R | GTGCTCCCCCGCCAATTCCT |  | (Stahl, 1991) |
|  | *apsA* | APS7-F | GGGYCTKTCCGCYATCAAYAC | 55 | (Friedrich, 2002) |
|  |  | apsA-R2 | CGACTTGAAGAGCAGGAACCAG |  | Unpublished |
|  | *mcrA* | MLf | GGTGGTGTMGGATTCACACARTAYGCWACAGC | 50 | (Luton et al., 2002) |
|  |  | MLr | TTCATTGCRTAGTTWGGRTAGTT |  | (Luton et al., 2002) |
|  | ANME-1 | ANME-1F | GCTTTCAGGGAATACTGC | 60 | (Lloyd et al., 2011) |
|  |  | ANME-1R | TCGCAGTAATGCCAACAC |  | (Lloyd et al., 2011) |
|  | ANME-2c | ANME-2cR | TCCTCTGGGAAATCTGGTTG | 60 | (Vigneron et al., 2013) |
|  |  | ANME-2cF | TCGTTTACGGCTGGGACTAC |  | (Vigneron et al., 2013) |
|  | ANME-3 | ANME-3F | GGATTGGCATAACACCGG | 60 | (Vigneron et al., 2013) |
|  |  | ANME-3R | TATGCTGGCACTCAGTGTCC |  | (Vigneron et al., 2013) |
| Sequencing | Bacteria | 338F | 5'- ACTCCTACGGGAGGCAGCAG-3' | - | (Mori et al., 2013) |
|  |  | 806R | 5'- GGACTACHVGGGTWTCTAAT-3' |  | (Mori et al., 2013) |
|  | Archaea | Arch344F | 5'-ACGGGGYGCAGCAGGCGCGA-3' | - | (Raskin et al., 1994) |
|  |  | Arch915R | 5'-GTGCTCCCCCGCCAATTCCT-3' |  | (Stahl, 1991) |

**Supplementary Table S2.** Analysis of differences between 16S rDNA and rRNA-derived sequences at different sites using Bray Curtis in the cold seeps.

| Community | Site | vs. | R | Significance |
| --- | --- | --- | --- | --- |
| Archaea | ROV05-02 | DNA vs. RNA | -0.04762 | 0.635 |
|  | ROV07-01 | DNA vs. RNA | **0.6793** | **0.003** |
|  | ROV07-02 | DNA vs. RNA | **0.6278** | **0.003** |
| Bacteria | ROV05-02 | DNA vs. RNA | **0.504** | **0.003** |
|  | ROV07-01 | DNA vs. RNA | **0.7182** | **0.001** |

**Supplementary Table S3.** Absolute abundance of bacteria, archaea, sulfate-reducing bacteria, and ANMEs, and the number of sequences and observed OTUs of archaea, methane-metabolizing archaea, and bacteria in the cold seeps.

| Sites | Depths (cm) | Bacteria (copies/g) | Archaea (copies/g) | *apsA* (copies/g) | *mcrA* (copies/g) | ANME-1 (copies/g) | ANME-2c (copies/g) | ANME-3 (copies/g) | 16S rDNA sequences | | | | | | 16S rRNA sequences | | | | | |
| --- | --- | --- | --- | --- | --- | --- | --- | --- | --- | --- | --- | --- | --- | --- | --- | --- | --- | --- | --- | --- |
|  |  |  |  |  |  |  |  |  | Archaea | | Methane-metabolizing archaea | | Bacteria | | Archaea | | Methane-metabolizing archaea | | Bacteria | |
|  |  |  |  |  |  |  |  |  | Sequence number (696,715) | OTU number (1,319) | Sequence number (357,023) | OTU number (104) | Sequence number (811,920) | OTU number (9,409) | Sequence number (367,333) | OTU number (919) | Sequence number (231,224) | OTU number (101) | Sequence number (674,266) | OTU number (7,083) |
| ROV05-02 | LC^a^ | 2.81E+07 | 3.75E+06 | 2.52E+06 | 2.14E+05 | 5.42E+05 | 8.22E+04 | 3.57E+06 | 44529 | 205 | 40562 | 31 | 42142 | 1270 | 9992 | 152 | 8294 | 20 | 37659 | 1037 |
|  | 0 | 4.14E+06 | 3.30E+06 | 3.72E+05 | 7.24E+04 | BD | BD | 3.61E+06 | 32268 | 103 | 31704 | 21 | 39905 | 1782 | 11647 | 72 | 11306 | 16 | 48961 | 1505 |
|  | 5 | 4.53E+06 | 2.42E+06 | 1.26E+06 | 3.18E+04 | BD | BD | 3.16E+06 | 27156 | 386 | 20107 | 60 | 38304 | 3055 | 10355 | 243 | 7322 | 45 | 65047 | 2494 |
|  | 15 | 4.68E+06 | 1.50E+06 | 2.42E+06 | ND^c^ | BD | BD | 5.07E+05 | 45267 | 430 | 34898 | 65 | 41516 | 3206 | 9851 | 194 | 6618 | 58 | 58978 | 1254 |
|  | 25 | 6.91E+06 | 3.74E+06 | 2.84E+06 | 7.55E+04 | BD | BD | 5.46E+05 | 36395 | 354 | 30020 | 58 | 33889 | 2454 | 10984 | 250 | 9201 | 59 | 58094 | 2540 |
|  | 35 | 6.84E+06 | 4.50E+06 | 4.34E+06 | 2.88E+05 | BD | BD | 2.58E+05 | 29059 | 243 | 26059 | 53 | 42821 | 2368 | 10154 | 154 | 9544 | 57 | 53237 | 2543 |
|  | 45 | 1.00E+07 | 1.49E+07 | 1.06E+07 | 3.00E+05 | 1.35E+05 | BD | 3.22E+05 | 32800 | 283 | 26304 | 65 | 42216 | 2543 | 12956 | 197 | 7920 | 61 | 70241 | 1477 |
| ROV07-01 | CS^b^ | 7.17E+07 | 1.38E+07 | 2.75E+07 | 9.07E+05 | 4.01E+06 | 3.70E+07 | 3.32E+06 | 35557 | 243 | 32263 | 39 | 45450 | 1791 | 24820 | 120 | 23978 | 27 | 22543 | 1128 |
|  | 0 | 5.25E+07 | 7.46E+06 | 2.20E+06 | BD^d^ | 1.78E+07 | 4.57E+05 | 1.52E+05 | 25245 | 290 | 10633 | 40 | 43240 | 2568 | 33059 | 351 | 16661 | 37 | 29183 | 1829 |
|  | 5 | 1.53E+07 | 9.76E+06 | 5.36E+06 | 7.19E+05 | 2.56E+07 | 3.64E+06 | 1.26E+06 | 30726 | 193 | 16399 | 32 | 44031 | 2066 | 25213 | 119 | 21722 | 27 | 43896 | 1532 |
|  | 15 | 8.52E+06 | 6.11E+06 | 2.40E+06 | 2.12E+05 | 3.04E+07 | 1.97E+06 | 8.83E+05 | 42302 | 287 | 23755 | 35 | 43529 | 2191 | 25791 | 111 | 22896 | 23 | 49307 | 1787 |
|  | 25 | 4.44E+06 | 1.14E+06 | 1.06E+06 | 8.81E+04 | BD | 3.74E+03 | BD | 41481 | 388 | 25819 | 36 | 38111 | 1917 | 34036 | 140 | 29631 | 31 | 44905 | 1799 |
|  | 35 | 3.11E+06 | 1.59E+06 | 1.17E+06 | 1.36E+05 | 3.99E+07 | 5.27E+06 | 1.09E+06 | 26150 | 249 | 18628 | 34 | 35169 | 1507 | 31390 | 195 | 29293 | 36 | 38454 | 1532 |
|  | 45 | 3.85E+06 | 1.71E+06 | 4.86E+05 | 1.65E+05 | 2.88E+07 | 1.64E+07 | 1.41E+06 | 28120 | 282 | 17393 | 35 | 36844 | 1661 | 23671 | 113 | 18969 | 25 | 53761 | 1895 |
| ROV07-02 | 0 | 1.84E+07 | 5.77E+06 | ND | BD | BD | BD | BD | 37091 | 442 | 212 | 13 | 45559 | 3592 | 14262 | 416 | 242 | 11 | ND | ND |
|  | 5 | 5.34E+07 | 1.08E+07 | ND | ND | BD | BD | BD | 25448 | 511 | 341 | 12 | 47459 | 3580 | 15817 | 437 | 848 | 10 | ND | ND |
|  | 15 | 2.70E+07 | 5.18E+06 | ND | BD | BD | BD | BD | 29133 | 458 | 553 | 10 | 39860 | 3337 | 17973 | 355 | 2768 | 12 | ND | ND |
|  | 25 | 6.11E+06 | 8.34E+05 | ND | ND | BD | BD | BD | 50622 | 452 | 400 | 8 | 38108 | 2829 | 14118 | 252 | 1618 | 8 | ND | ND |
|  | 35 | 7.17E+06 | 2.46E+06 | ND | BD | 1.75E+06 | BD | BD | 35806 | 470 | 407 | 14 | 37971 | 2413 | 16124 | 149 | 1117 | 4 | ND | ND |
|  | 45 | 5.46E+06 | 4.07E+06 | ND | BD | BD | BD | BD | 41560 | 378 | 566 | 15 | 35796 | 2403 | 15120 | 160 | 1276 | 8 | ND | ND |

^a^: LC represents the sediment sample with living clams at surface sediment

^b^: CS represents the sediment sample with die-off clam shells at surface sediment

^c^: ND represents none determined

^d^: BD refers to below detection limit

**Supplementary Table S4****.** Marginal tests from RDA showing the proportional contributions of different environmental factors to archaeal community distribution in the sediments of cold seeps.

| Variable | P | Prop. |
| --- | --- | --- |
| Depth | 0.675 | 4.17E-02 |
| **CH_4_** | **0.014** | **0.2111** |
| **SO_4_^2-^** | **0.004** | **0.25425** |
| Cl^-^ | 0.295 | 8.44E-02 |
| **Ca^2+^** | **0.001** | **0.26327** |
| Mg^2+^ | 0.597 | 5.21E-02 |
| K^+^ | 0.759 | 3.47E-02 |
| Na^+^ | 0.634 | 4.27E-02 |
| **TA** | **0.006** | **0.22198** |
| **DIC** | **0.005** | **0.19235** |
| **δ^13^C_DIC_** | **0.002** | **0.22823** |
| Sand | 0.794 | 2.95E-02 |
| Silt | 0.672 | 3.96E-02 |
| Clay | 0.787 | 3.10E-02 |

Significant correlations are in bold (P<0.05); Abbreviations: Prop., proportional contribution

**Supplementary Table S5.** The list of archaeal and bacterial OTUs correlated with ANME-2dI and ANME-2a/b in networks.

| The OTUs correlated with ANME-2dI and ANME-2a/b | ANMEs | Sites |
| --- | --- | --- |
| d__Archaea;k__norank_d__Archaea;p__Asgardaeota;c__Lokiarchaeia;o__norank_c__Lokiarchaeia;f__norank_c__Lokiarchaeia;g__norank_c__Lokiarchaeia;s__uncultured_archaeon_g__norank_c__Lokiarchaeia;OTU1424 | ANME-2dI | ROV05-02 |
| d__Archaea;k__norank_d__Archaea;p__Asgardaeota;c__Lokiarchaeia;o__norank_c__Lokiarchaeia;f__norank_c__Lokiarchaeia;g__norank_c__Lokiarchaeia;s__uncultured_archaeon_g__norank_c__Lokiarchaeia;OTU349 | ANME-2dI | ROV05-02 |
| d__Archaea;k__norank_d__Archaea;p__Asgardaeota;c__Lokiarchaeia;o__norank_c__Lokiarchaeia;f__norank_c__Lokiarchaeia;g__norank_c__Lokiarchaeia;s__uncultured_archaeon_g__norank_c__Lokiarchaeia;OTU700 | ANME-2dI | ROV05-02 |
| d__Archaea;k__norank_d__Archaea;p__Asgardaeota;c__Lokiarchaeia;o__norank_c__Lokiarchaeia;f__norank_c__Lokiarchaeia;g__norank_c__Lokiarchaeia;s__uncultured_archaeon_g__norank_c__Lokiarchaeia;OTU901 | ANME-2dI | ROV05-02 |
| d__Archaea;k__norank_d__Archaea;p__Asgardaeota;c__Lokiarchaeia;o__norank_c__Lokiarchaeia;f__norank_c__Lokiarchaeia;g__norank_c__Lokiarchaeia;s__uncultured_archaeon_g__norank_c__Lokiarchaeia;OTU938 | ANME-2dI | ROV05-02 |
| d__Archaea;k__norank_d__Archaea;p__Asgardaeota;c__unclassified_p__Asgardaeota;o__unclassified_p__Asgardaeota;f__unclassified_p__Asgardaeota;g__unclassified_p__Asgardaeota;s__unclassified_p__Asgardaeota;OTU913 | ANME-2a/b | ROV07-01 |
| d__Archaea;k__norank_d__Archaea;p__Crenarchaeota;c__Bathyarchaeia;o__norank_c__Bathyarchaeia;f__norank_c__Bathyarchaeia;g__norank_c__Bathyarchaeia;s__marine_metagenome_g__norank_c__Bathyarchaeia;OTU1161 | ANME-2dI | ROV05-02 |
| d__Archaea;k__norank_d__Archaea;p__Crenarchaeota;c__Bathyarchaeia;o__norank_c__Bathyarchaeia;f__norank_c__Bathyarchaeia;g__norank_c__Bathyarchaeia;s__marine_metagenome_g__norank_c__Bathyarchaeia;OTU205 | ANME-2dI | ROV05-02 |
| d__Archaea;k__norank_d__Archaea;p__Crenarchaeota;c__Bathyarchaeia;o__norank_c__Bathyarchaeia;f__norank_c__Bathyarchaeia;g__norank_c__Bathyarchaeia;s__miscellaneous_Crenarchaeota_group_archaeon_SMTZ1-55;OTU911 | ANME-2dI | ROV05-02 |
| d__Archaea;k__norank_d__Archaea;p__Euryarchaeota;c__Methanomicrobia;o__Methanosarcinales;f__ANME-2a-2b;g__norank_f__ANME-2a-2b;s__uncultured_ANME-2_cluster_archaeon_g__norank_f__ANME-2a-2b;OTU1014 | ANME-2a/b | ROV07-01 |
| d__Archaea;k__norank_d__Archaea;p__Euryarchaeota;c__Methanomicrobia;o__Methanosarcinales;f__ANME-2a-2b;g__norank_f__ANME-2a-2b;s__uncultured_ANME-2_cluster_archaeon_g__norank_f__ANME-2a-2b;OTU1048 | ANME-2a/b | ROV07-01 |
| d__Archaea;k__norank_d__Archaea;p__Euryarchaeota;c__Methanomicrobia;o__Methanosarcinales;f__ANME-2a-2b;g__norank_f__ANME-2a-2b;s__uncultured_ANME-2_cluster_archaeon_g__norank_f__ANME-2a-2b;OTU661 | ANME-2a/b | ROV07-01 |
| d__Archaea;k__norank_d__Archaea;p__Euryarchaeota;c__Methanomicrobia;o__Methanosarcinales;f__ANME-2a-2b;g__norank_f__ANME-2a-2b;s__uncultured_ANME-2_cluster_archaeon_g__norank_f__ANME-2a-2b;OTU804 | ANME-2a/b | ROV07-01 |
| d__Archaea;k__norank_d__Archaea;p__Euryarchaeota;c__Methanomicrobia;o__Methanosarcinales;f__ANME-2a-2b;g__norank_f__ANME-2a-2b;s__uncultured_ANME-2_cluster_archaeon_g__norank_f__ANME-2a-2b;OTU940 | ANME-2a/b | ROV05-02 |
| d__Archaea;k__norank_d__Archaea;p__Euryarchaeota;c__Methanomicrobia;o__Methanosarcinales;f__ANME-2a-2b;g__norank_f__ANME-2a-2b;s__uncultured_ANME-2_cluster_archaeon_g__norank_f__ANME-2a-2b;OTU940 | ANME-2a/b | ROV07-01 |
| d__Archaea;k__norank_d__Archaea;p__Euryarchaeota;c__Methanomicrobia;o__Methanosarcinales;f__ANME-2a-2b;g__norank_f__ANME-2a-2b;s__uncultured_archaeon_g__norank_f__ANME-2a-2b;OTU702 | ANME-2a/b | ROV07-01 |
| d__Archaea;k__norank_d__Archaea;p__Euryarchaeota;c__Methanomicrobia;o__Methanosarcinales;f__Methanoperedenaceae;g__norank_f__Methanoperedenaceae;s__uncultured_archaeon_g__norank_f__Methanoperedenaceae;OTU905 | ANME-2a/b | ROV07-01 |
| d__Archaea;k__norank_d__Archaea;p__Euryarchaeota;c__Methanomicrobia;o__Methanosarcinales;f__Methanosarcinaceae;g__Methanococcoides;s__uncultured_archaeon_g__Methanococcoides;OTU952 | ANME-2a/b | ROV07-01 |
| d__Archaea;k__norank_d__Archaea;p__Hydrothermarchaeota;c__norank_p__Hydrothermarchaeota;o__norank_p__Hydrothermarchaeota;f__norank_p__Hydrothermarchaeota;g__norank_p__Hydrothermarchaeota;s__unclassified_g__norank_p__Hydrothermarchaeota;OTU146 | ANME-2dI | ROV05-02 |
| d__Archaea;k__norank_d__Archaea;p__Thaumarchaeota;c__Marine_Benthic_Group_A;o__norank_c__Marine_Benthic_Group_A;f__norank_c__Marine_Benthic_Group_A;g__norank_c__Marine_Benthic_Group_A;s__uncultured_archaeon_g__norank_c__Marine_Benthic_Group_A;OTU189 | ANME-2dI | ROV05-02 |
| d__Archaea;k__norank_d__Archaea;p__unclassified_k__norank_d__Archaea;c__unclassified_k__norank_d__Archaea;o__unclassified_k__norank_d__Archaea;f__unclassified_k__norank_d__Archaea;g__unclassified_k__norank_d__Archaea;s__unclassified_k__norank_d__Archaea;OTU936 | ANME-2dI | ROV05-02 |
| d__Archaea;k__norank_d__Archaea;p__unclassified_k__norank_d__Archaea;c__unclassified_k__norank_d__Archaea;o__unclassified_k__norank_d__Archaea;f__unclassified_k__norank_d__Archaea;g__unclassified_k__norank_d__Archaea;s__unclassified_k__norank_d__Archaea;OTU513 | ANME-2a/b | ROV05-02 |
| d__Bacteria;k__norank_d__Bacteria;p__Acidobacteria;c__Subgroup_21;o__norank_c__Subgroup_21;f__norank_c__Subgroup_21;g__norank_c__Subgroup_21;s__uncultured_bacterium_g__norank_c__Subgroup_21;OTU10227 | ANME-2dI | ROV05-02 |
| d__Bacteria;k__norank_d__Bacteria;p__Acidobacteria;c__Subgroup_9;o__norank_c__Subgroup_9;f__norank_c__Subgroup_9;g__norank_c__Subgroup_9;s__uncultured_bacterium_g__norank_c__Subgroup_9;OTU1340 | ANME-2dI | ROV05-02 |
| d__Bacteria;k__norank_d__Bacteria;p__Acidobacteria;c__Thermoanaerobaculia;o__Thermoanaerobaculales;f__Thermoanaerobaculaceae;g__Subgroup_10;s__unclassified_g__Subgroup_10;OTU9205 | ANME-2dI | ROV05-02 |
| d__Bacteria;k__norank_d__Bacteria;p__Acidobacteria;c__Thermoanaerobaculia;o__Thermoanaerobaculales;f__Thermoanaerobaculaceae;g__Subgroup_10;s__uncultured_Acidobacteriaceae_bacterium_g__Subgroup_10;OTU3871 | ANME-2dI | ROV05-02 |
| d__Bacteria;k__norank_d__Bacteria;p__Acidobacteria;c__Thermoanaerobaculia;o__Thermoanaerobaculales;f__Thermoanaerobaculaceae;g__Subgroup_23;s__uncultured_bacterium_g__Subgroup_23;OTU7667 | ANME-2a/b | ROV07-01 |
| d__Bacteria;k__norank_d__Bacteria;p__Acidobacteria;c__Thermoanaerobaculia;o__Thermoanaerobaculales;f__Thermoanaerobaculaceae;g__Subgroup_23;s__uncultured_bacterium_g__Subgroup_23;OTU9349 | ANME-2dI | ROV05-02 |
| d__Bacteria;k__norank_d__Bacteria;p__Actinobacteria;c__Actinobacteria;o__Actinomarinales;f__norank_o__Actinomarinales;g__norank_o__Actinomarinales;s__marine_metagenome_g__norank;OTU9371 | ANME-2a/b | ROV05-02 |
| d__Bacteria;k__norank_d__Bacteria;p__Actinobacteria;c__Actinobacteria;o__Actinomarinales;f__norank_o__Actinomarinales;g__norank_o__Actinomarinales;s__marine_metagenome_g__norank;OTU9371 | ANME-2a/b | ROV05-02 |
| d__Bacteria;k__norank_d__Bacteria;p__Actinobacteria;c__Actinobacteria;o__Actinomarinales;f__norank_o__Actinomarinales;g__norank_o__Actinomarinales;s__uncultured_bacterium_g__norank_o__Actinomarinales;OTU8015 | ANME-2a/b | ROV05-02 |
| d__Bacteria;k__norank_d__Bacteria;p__Actinobacteria;c__Actinobacteria;o__Actinomarinales;f__norank_o__Actinomarinales;g__norank_o__Actinomarinales;s__uncultured_bacterium_g__norank_o__Actinomarinales;OTU8015 | ANME-2a/b | ROV05-02 |
| d__Bacteria;k__norank_d__Bacteria;p__Actinobacteria;c__Actinobacteria;o__Actinomarinales;f__norank_o__Actinomarinales;g__norank_o__Actinomarinales;s__uncultured_bacterium_g__norank_o__Actinomarinales;OTU8015 | ANME-2a/b | ROV07-01 |
| d__Bacteria;k__norank_d__Bacteria;p__Actinobacteria;c__Actinobacteria;o__Actinomarinales;f__norank_o__Actinomarinales;g__norank_o__Actinomarinales;s__uncultured_Gram-positive_bacterium_g__norank_o__Actinomarinales;OTU9184 | ANME-2a/b | ROV05-02 |
| d__Bacteria;k__norank_d__Bacteria;p__Actinobacteria;c__Actinobacteria;o__Actinomarinales;f__norank_o__Actinomarinales;g__norank_o__Actinomarinales;s__uncultured_Gram-positive_bacterium_g__norank_o__Actinomarinales;OTU9184 | ANME-2a/b | ROV05-02 |
| d__Bacteria;k__norank_d__Bacteria;p__Actinobacteria;c__Actinobacteria;o__norank_c__Actinobacteria;f__norank_c__Actinobacteria;g__norank_c__Actinobacteria;s__uncultured_organism_g__norank_c__Actinobacteria;OTU8731 | ANME-2dI | ROV05-02 |
| d__Bacteria;k__norank_d__Bacteria;p__Actinobacteria;c__Actinobacteria;o__norank_c__Actinobacteria;f__norank_c__Actinobacteria;g__norank_c__Actinobacteria;s__uncultured_organism_g__norank_c__Actinobacteria;OTU8731 | ANME-2a/b | ROV07-01 |
| d__Bacteria;k__norank_d__Bacteria;p__Aegiribacteria;c__norank_p__Aegiribacteria;o__norank_p__Aegiribacteria;f__norank_p__Aegiribacteria;g__norank_p__Aegiribacteria;s__uncultured_bacterium_g__norank_p__Aegiribacteria;OTU6710 | ANME-2a/b | ROV07-01 |
| d__Bacteria;k__norank_d__Bacteria;p__Bacteroidetes;c__Bacteroidia;o__Bacteroidales;f__Bacteroidetes_BD2-2;g__norank_f__Bacteroidetes_BD2-2;s__unclassified_g__norank_f__Bacteroidetes_BD2-2;OTU4707 | ANME-2a/b | ROV05-02 |
| d__Bacteria;k__norank_d__Bacteria;p__Bacteroidetes;c__Bacteroidia;o__Bacteroidales;f__Bacteroidetes_BD2-2;g__norank_f__Bacteroidetes_BD2-2;s__unclassified_g__norank_f__Bacteroidetes_BD2-2;OTU4707 | ANME-2a/b | ROV05-02 |
| d__Bacteria;k__norank_d__Bacteria;p__Bacteroidetes;c__Bacteroidia;o__Bacteroidales;f__Bacteroidetes_BD2-2;g__norank_f__Bacteroidetes_BD2-2;s__unclassified_g__norank_f__Bacteroidetes_BD2-2;OTU4707 | ANME-2a/b | ROV07-01 |
| d__Bacteria;k__norank_d__Bacteria;p__Bacteroidetes;c__Bacteroidia;o__Bacteroidales;f__Marinilabiliaceae;g__Labilibacter;s__uncultured_Bacteroidetes_bacterium_g__Labilibacter;OTU4970 | ANME-2a/b | ROV05-02 |
| d__Bacteria;k__norank_d__Bacteria;p__Bacteroidetes;c__Bacteroidia;o__Bacteroidales;f__Marinilabiliaceae;g__Labilibacter;s__uncultured_Bacteroidetes_bacterium_g__Labilibacter;OTU4970 | ANME-2a/b | ROV05-02 |
| d__Bacteria;k__norank_d__Bacteria;p__Bacteroidetes;c__Bacteroidia;o__Bacteroidales;f__Prolixibacteraceae;g__Draconibacterium;s__unclassified_g__Draconibacterium;OTU8310 | ANME-2a/b | ROV05-02 |
| d__Bacteria;k__norank_d__Bacteria;p__Bacteroidetes;c__Bacteroidia;o__Bacteroidales;f__Prolixibacteraceae;g__Draconibacterium;s__unclassified_g__Draconibacterium;OTU8310 | ANME-2a/b | ROV05-02 |
| d__Bacteria;k__norank_d__Bacteria;p__Bacteroidetes;c__Bacteroidia;o__Bacteroidales;f__Prolixibacteraceae;g__Draconibacterium;s__unclassified_g__Draconibacterium;OTU8310 | ANME-2a/b | ROV07-01 |
| d__Bacteria;k__norank_d__Bacteria;p__Bacteroidetes;c__Bacteroidia;o__Bacteroidales;f__Prolixibacteraceae;g__Draconibacterium;s__uncultured_bacterium_g__Draconibacterium;OTU4704 | ANME-2a/b | ROV05-02 |
| d__Bacteria;k__norank_d__Bacteria;p__Bacteroidetes;c__Bacteroidia;o__Bacteroidales;f__Prolixibacteraceae;g__Draconibacterium;s__uncultured_bacterium_g__Draconibacterium;OTU4704 | ANME-2a/b | ROV05-02 |
| d__Bacteria;k__norank_d__Bacteria;p__Bacteroidetes;c__Bacteroidia;o__Bacteroidetes_VC2.1_Bac22;f__norank_o__Bacteroidetes_VC2.1_Bac22;g__norank_o__Bacteroidetes_VC2.1_Bac22;s__unclassified_g__norank_o__Bacteroidetes_VC2.1_Bac22;OTU8039 | ANME-2a/b | ROV07-01 |
| d__Bacteria;k__norank_d__Bacteria;p__Bacteroidetes;c__Bacteroidia;o__Bacteroidetes_VC2.1_Bac22;f__norank_o__Bacteroidetes_VC2.1_Bac22;g__norank_o__Bacteroidetes_VC2.1_Bac22;s__uncultured_Bacteroidetes_bacterium_g__norank_o__Bacteroidetes_VC2.1_Bac22;OTU8163 | ANME-2a/b | ROV07-01 |
| d__Bacteria;k__norank_d__Bacteria;p__Bacteroidetes;c__Bacteroidia;o__Flavobacteriales;f__Flavobacteriaceae;g__Lutibacter;s__uncultured_bacterium_g__Lutibacter;OTU4717 | ANME-2a/b | ROV05-02 |
| d__Bacteria;k__norank_d__Bacteria;p__Bacteroidetes;c__Bacteroidia;o__Flavobacteriales;f__Flavobacteriaceae;g__Lutibacter;s__uncultured_bacterium_g__Lutibacter;OTU4717 | ANME-2a/b | ROV05-02 |
| d__Bacteria;k__norank_d__Bacteria;p__Bacteroidetes;c__Bacteroidia;o__Flavobacteriales;f__Flavobacteriaceae;g__Lutimonas;s__uncultured_Bacteroidetes_bacterium_g__Lutimonas;OTU5499 | ANME-2a/b | ROV07-01 |
| d__Bacteria;k__norank_d__Bacteria;p__Bacteroidetes;c__Bacteroidia;o__Flavobacteriales;f__Flavobacteriaceae;g__norank_f__Flavobacteriaceae;s__Bacteroidetes_bacterium_ectosymbiont_of_Rimicaris_exoculata_g__norank;OTU8223 | ANME-2a/b | ROV07-01 |
| d__Bacteria;k__norank_d__Bacteria;p__Bacteroidetes;c__Bacteroidia;o__Sphingobacteriales;f__Lentimicrobiaceae;g__norank_f__Lentimicrobiaceae;s__unclassified_g__norank_f__Lentimicrobiaceae;OTU8218 | ANME-2a/b | ROV07-01 |
| d__Bacteria;k__norank_d__Bacteria;p__Bacteroidetes;c__Ignavibacteria;o__Ignavibacteriales;f__PHOS-HE36;g__norank_f__PHOS-HE36;s__uncultured_Chlorobi_bacterium_g__norank_f__PHOS-HE36;OTU8790 | ANME-2dI | ROV05-02 |
| d__Bacteria;k__norank_d__Bacteria;p__Bacteroidetes;c__Ignavibacteria;o__Ignavibacteriales;f__PHOS-HE36;g__norank_f__PHOS-HE36;s__uncultured_deep-sea_bacterium_g__norank_f__PHOS-HE36;OTU4541 | ANME-2dI | ROV05-02 |
| d__Bacteria;k__norank_d__Bacteria;p__Calditrichaeota;c__Calditrichia;o__Calditrichales;f__Calditrichaceae;g__norank_f__Calditrichaceae;s__uncultured_Deferribacteres_bacterium_g__norank_f__Calditrichaceae;OTU7800 | ANME-2dI | ROV05-02 |
| d__Bacteria;k__norank_d__Bacteria;p__Chloroflexi;c__Anaerolineae;o__Anaerolineales;f__Anaerolineaceae;g__norank_f__Anaerolineaceae;s__unclassified_g__norank_f__Anaerolineaceae;OTU8532 | ANME-2a/b | ROV05-02 |
| d__Bacteria;k__norank_d__Bacteria;p__Chloroflexi;c__Anaerolineae;o__Anaerolineales;f__Anaerolineaceae;g__norank_f__Anaerolineaceae;s__unclassified_g__norank_f__Anaerolineaceae;OTU8532 | ANME-2a/b | ROV05-02 |
| d__Bacteria;k__norank_d__Bacteria;p__Chloroflexi;c__Anaerolineae;o__Anaerolineales;f__Anaerolineaceae;g__norank_f__Anaerolineaceae;s__unclassified_g__norank_f__Anaerolineaceae;OTU8532 | ANME-2a/b | ROV07-01 |
| d__Bacteria;k__norank_d__Bacteria;p__Chloroflexi;c__Anaerolineae;o__Anaerolineales;f__Anaerolineaceae;g__norank_f__Anaerolineaceae;s__uncultured_Chloroflexi_bacterium_g__norank_f__Anaerolineaceae;OTU8187 | ANME-2a/b | ROV05-02 |
| d__Bacteria;k__norank_d__Bacteria;p__Chloroflexi;c__Anaerolineae;o__Anaerolineales;f__Anaerolineaceae;g__norank_f__Anaerolineaceae;s__uncultured_Chloroflexi_bacterium_g__norank_f__Anaerolineaceae;OTU8187 | ANME-2a/b | ROV05-02 |
| d__Bacteria;k__norank_d__Bacteria;p__Chloroflexi;c__Anaerolineae;o__Anaerolineales;f__Anaerolineaceae;g__norank_f__Anaerolineaceae;s__uncultured_marine_bacterium_g__norank_f__Anaerolineaceae;OTU9765 | ANME-2a/b | ROV07-01 |
| d__Bacteria;k__norank_d__Bacteria;p__Chloroflexi;c__Anaerolineae;o__Anaerolineales;f__Anaerolineaceae;g__norank_f__Anaerolineaceae;s__uncultured_organism_g__norank_f__Anaerolineaceae;OTU5268 | ANME-2a/b | ROV05-02 |
| d__Bacteria;k__norank_d__Bacteria;p__Chloroflexi;c__Anaerolineae;o__Anaerolineales;f__Anaerolineaceae;g__norank_f__Anaerolineaceae;s__uncultured_organism_g__norank_f__Anaerolineaceae;OTU5268 | ANME-2a/b | ROV05-02 |
| d__Bacteria;k__norank_d__Bacteria;p__Chloroflexi;c__Anaerolineae;o__Anaerolineales;f__Anaerolineaceae;g__norank_f__Anaerolineaceae;s__uncultured_organism_g__norank_f__Anaerolineaceae;OTU5268 | ANME-2a/b | ROV07-01 |
| d__Bacteria;k__norank_d__Bacteria;p__Chloroflexi;c__Anaerolineae;o__Anaerolineales;f__Anaerolineaceae;g__Pelolinea;s__uncultured_bacterium_g__Pelolinea;OTU8089 | ANME-2a/b | ROV07-01 |
| d__Bacteria;k__norank_d__Bacteria;p__Chloroflexi;c__Anaerolineae;o__SBR1031;f__norank_o__SBR1031;g__norank_o__SBR1031;s__unclassified_g__norank_o__SBR1031;OTU8014 | ANME-2a/b | ROV07-01 |
| d__Bacteria;k__norank_d__Bacteria;p__Chloroflexi;c__Anaerolineae;o__SJA-15;f__norank_o__SJA-15;g__norank_o__SJA-15;s__uncultured_bacterium_g__norank_o__SJA-15;OTU8098 | ANME-2a/b | ROV07-01 |
| d__Bacteria;k__norank_d__Bacteria;p__Chloroflexi;c__Dehalococcoidia;o__S085;f__norank_o__S085;g__norank_o__S085;s__unclassified_g__norank_o__S085;OTU3584 | ANME-2dI | ROV05-02 |
| d__Bacteria;k__norank_d__Bacteria;p__Chloroflexi;c__Dehalococcoidia;o__S085;f__norank_o__S085;g__norank_o__S085;s__uncultured_bacterium_g__norank_o__S085;OTU1282 | ANME-2dI | ROV05-02 |
| d__Bacteria;k__norank_d__Bacteria;p__Chloroflexi;c__Dehalococcoidia;o__S085;f__norank_o__S085;g__norank_o__S085;s__uncultured_bacterium_g__norank_o__S085;OTU3077 | ANME-2dI | ROV05-02 |
| d__Bacteria;k__norank_d__Bacteria;p__Chloroflexi;c__Dehalococcoidia;o__S085;f__norank_o__S085;g__norank_o__S085;s__uncultured_bacterium_g__norank_o__S085;OTU3095 | ANME-2dI | ROV05-02 |
| d__Bacteria;k__norank_d__Bacteria;p__Chloroflexi;c__Dehalococcoidia;o__S085;f__norank_o__S085;g__norank_o__S085;s__uncultured_bacterium_g__norank_o__S085;OTU3115 | ANME-2dI | ROV05-02 |
| d__Bacteria;k__norank_d__Bacteria;p__Chloroflexi;c__Dehalococcoidia;o__S085;f__norank_o__S085;g__norank_o__S085;s__uncultured_bacterium_g__norank_o__S085;OTU883 | ANME-2dI | ROV05-02 |
| d__Bacteria;k__norank_d__Bacteria;p__Chloroflexi;c__Dehalococcoidia;o__S085;f__norank_o__S085;g__norank_o__S085;s__uncultured_bacterium_g__norank_o__S085;OTU929 | ANME-2dI | ROV05-02 |
| d__Bacteria;k__norank_d__Bacteria;p__Chloroflexi;c__Dehalococcoidia;o__S085;f__norank_o__S085;g__norank_o__S085;s__uncultured_bacterium_g__norank_o__S085;OTU999 | ANME-2dI | ROV05-02 |
| d__Bacteria;k__norank_d__Bacteria;p__Chloroflexi;c__JG30-KF-CM66;o__norank_c__JG30-KF-CM66;f__norank_c__JG30-KF-CM66;g__norank_c__JG30-KF-CM66;s__uncultured_bacterium_g__norank_c__JG30-KF-CM66;OTU2284 | ANME-2dI | ROV05-02 |
| d__Bacteria;k__norank_d__Bacteria;p__Chloroflexi;c__JG30-KF-CM66;o__norank_c__JG30-KF-CM66;f__norank_c__JG30-KF-CM66;g__norank_c__JG30-KF-CM66;s__uncultured_bacterium_g__norank_c__JG30-KF-CM66;OTU2291 | ANME-2dI | ROV05-02 |
| d__Bacteria;k__norank_d__Bacteria;p__Chloroflexi;c__JG30-KF-CM66;o__norank_c__JG30-KF-CM66;f__norank_c__JG30-KF-CM66;g__norank_c__JG30-KF-CM66;s__uncultured_bacterium_g__norank_c__JG30-KF-CM66;OTU2293 | ANME-2dI | ROV05-02 |
| d__Bacteria;k__norank_d__Bacteria;p__Chloroflexi;c__JG30-KF-CM66;o__norank_c__JG30-KF-CM66;f__norank_c__JG30-KF-CM66;g__norank_c__JG30-KF-CM66;s__uncultured_bacterium_g__norank_c__JG30-KF-CM66;OTU2306 | ANME-2dI | ROV05-02 |
| d__Bacteria;k__norank_d__Bacteria;p__Chloroflexi;c__JG30-KF-CM66;o__norank_c__JG30-KF-CM66;f__norank_c__JG30-KF-CM66;g__norank_c__JG30-KF-CM66;s__uncultured_bacterium_g__norank_c__JG30-KF-CM66;OTU2320 | ANME-2dI | ROV05-02 |
| d__Bacteria;k__norank_d__Bacteria;p__Chloroflexi;c__JG30-KF-CM66;o__norank_c__JG30-KF-CM66;f__norank_c__JG30-KF-CM66;g__norank_c__JG30-KF-CM66;s__uncultured_bacterium_g__norank_c__JG30-KF-CM66;OTU8839 | ANME-2dI | ROV05-02 |
| d__Bacteria;k__norank_d__Bacteria;p__Chloroflexi;c__JG30-KF-CM66;o__norank_c__JG30-KF-CM66;f__norank_c__JG30-KF-CM66;g__norank_c__JG30-KF-CM66;s__uncultured_bacterium_g__norank_c__JG30-KF-CM66;OTU8873 | ANME-2dI | ROV05-02 |
| d__Bacteria;k__norank_d__Bacteria;p__Chloroflexi;c__JG30-KF-CM66;o__norank_c__JG30-KF-CM66;f__norank_c__JG30-KF-CM66;g__norank_c__JG30-KF-CM66;s__uncultured_bacterium_g__norank_c__JG30-KF-CM66;OTU9933 | ANME-2dI | ROV05-02 |
| d__Bacteria;k__norank_d__Bacteria;p__Chloroflexi;c__KD4-96;o__norank_c__KD4-96;f__norank_c__KD4-96;g__norank_c__KD4-96;s__uncultured_bacterium_g__norank_c__KD4-96;OTU3142 | ANME-2a/b | ROV05-02 |
| d__Bacteria;k__norank_d__Bacteria;p__Chloroflexi;c__KD4-96;o__norank_c__KD4-96;f__norank_c__KD4-96;g__norank_c__KD4-96;s__uncultured_bacterium_g__norank_c__KD4-96;OTU3142 | ANME-2a/b | ROV05-02 |
| d__Bacteria;k__norank_d__Bacteria;p__Dadabacteria;c__Dadabacteriia;o__Dadabacteriales;f__norank_o__Dadabacteriales;g__norank_o__Dadabacteriales;s__unclassified_g__norank_o__Dadabacteriales;OTU6093 | ANME-2dI | ROV05-02 |
| d__Bacteria;k__norank_d__Bacteria;p__Dadabacteria;c__Dadabacteriia;o__Dadabacteriales;f__norank_o__Dadabacteriales;g__norank_o__Dadabacteriales;s__uncultured_bacterium_g__norank_o__Dadabacteriales;OTU1314 | ANME-2dI | ROV05-02 |
| d__Bacteria;k__norank_d__Bacteria;p__Dependentiae;c__Babeliae;o__Babeliales;f__Vermiphilaceae;g__norank_f__Vermiphilaceae;s__uncultured_Candidatus_Dependentiae_bacterium_g__norank;OTU9177 | ANME-2dI | ROV05-02 |
| d__Bacteria;k__norank_d__Bacteria;p__Epsilonbacteraeota;c__Campylobacteria;o__Campylobacterales;f__Sulfurovaceae;g__Sulfurovum;s__unclassified_g__Sulfurovum;OTU8138 | ANME-2a/b | ROV05-02 |
| d__Bacteria;k__norank_d__Bacteria;p__Epsilonbacteraeota;c__Campylobacteria;o__Campylobacterales;f__Sulfurovaceae;g__Sulfurovum;s__unclassified_g__Sulfurovum;OTU8138 | ANME-2a/b | ROV05-02 |
| d__Bacteria;k__norank_d__Bacteria;p__Epsilonbacteraeota;c__Campylobacteria;o__Campylobacterales;f__Sulfurovaceae;g__Sulfurovum;s__uncultured_proteobacterium_g__Sulfurovum;OTU4641 | ANME-2a/b | ROV05-02 |
| d__Bacteria;k__norank_d__Bacteria;p__Epsilonbacteraeota;c__Campylobacteria;o__Campylobacterales;f__Sulfurovaceae;g__Sulfurovum;s__uncultured_proteobacterium_g__Sulfurovum;OTU4641 | ANME-2a/b | ROV05-02 |
| d__Bacteria;k__norank_d__Bacteria;p__Epsilonbacteraeota;c__Campylobacteria;o__Campylobacterales;f__Sulfurovaceae;g__Sulfurovum;s__uncultured_proteobacterium_g__Sulfurovum;OTU4641 | ANME-2a/b | ROV07-01 |
| d__Bacteria;k__norank_d__Bacteria;p__Epsilonbacteraeota;c__Campylobacteria;o__Campylobacterales;f__Thiovulaceae;g__Sulfurimonas;s__unclassified_g__Sulfurimonas;OTU7785 | ANME-2a/b | ROV05-02 |
| d__Bacteria;k__norank_d__Bacteria;p__Epsilonbacteraeota;c__Campylobacteria;o__Campylobacterales;f__Thiovulaceae;g__Sulfurimonas;s__unclassified_g__Sulfurimonas;OTU7785 | ANME-2a/b | ROV05-02 |
| d__Bacteria;k__norank_d__Bacteria;p__Firmicutes;c__Clostridia;o__Clostridiales;f__Christensenellaceae;g__norank_f__Christensenellaceae;s__uncultured_bacterium_g__norank_f__Christensenellaceae;OTU160 | ANME-2a/b | ROV05-02 |
| d__Bacteria;k__norank_d__Bacteria;p__Firmicutes;c__Clostridia;o__Clostridiales;f__Christensenellaceae;g__norank_f__Christensenellaceae;s__uncultured_bacterium_g__norank_f__Christensenellaceae;OTU160 | ANME-2a/b | ROV05-02 |
| d__Bacteria;k__norank_d__Bacteria;p__Firmicutes;c__Clostridia;o__Clostridiales;f__Christensenellaceae;g__norank_f__Christensenellaceae;s__uncultured_bacterium_g__norank_f__Christensenellaceae;OTU160 | ANME-2a/b | ROV07-01 |
| d__Bacteria;k__norank_d__Bacteria;p__Firmicutes;c__Clostridia;o__Clostridiales;f__Lachnospiraceae;g__unclassified_f__Lachnospiraceae;s__unclassified_f__Lachnospiraceae;OTU8239 | ANME-2a/b | ROV07-01 |
| d__Bacteria;k__norank_d__Bacteria;p__Firmicutes;c__Clostridia;o__Clostridiales;f__Peptococcaceae;g__norank_f__Peptococcaceae;s__unclassified_g__norank_f__Peptococcaceae;OTU5818 | ANME-2a/b | ROV07-01 |
| d__Bacteria;k__norank_d__Bacteria;p__Firmicutes;c__Clostridia;o__Clostridiales;f__Ruminococcaceae;g__norank_f__Ruminococcaceae;s__unclassified_g__norank_f__Ruminococcaceae;OTU114 | ANME-2a/b | ROV05-02 |
| d__Bacteria;k__norank_d__Bacteria;p__Firmicutes;c__Clostridia;o__Clostridiales;f__Ruminococcaceae;g__norank_f__Ruminococcaceae;s__unclassified_g__norank_f__Ruminococcaceae;OTU114 | ANME-2a/b | ROV05-02 |
| d__Bacteria;k__norank_d__Bacteria;p__Fusobacteria;c__Fusobacteriia;o__Fusobacteriales;f__Fusobacteriaceae;g__Psychrilyobacter;s__uncultured_bacterium_g__Psychrilyobacter;OTU8058 | ANME-2a/b | ROV07-01 |
| d__Bacteria;k__norank_d__Bacteria;p__Gemmatimonadetes;c__Gemmatimonadetes;o__Gemmatimonadales;f__Gemmatimonadaceae;g__norank_f__Gemmatimonadaceae;s__unclassified_g__norank_f__Gemmatimonadaceae;OTU2247 | ANME-2dI | ROV05-02 |
| d__Bacteria;k__norank_d__Bacteria;p__Gemmatimonadetes;c__Gemmatimonadetes;o__Gemmatimonadales;f__Gemmatimonadaceae;g__norank_f__Gemmatimonadaceae;s__uncultured_bacterium_g__norank_f__Gemmatimonadaceae;OTU3117 | ANME-2dI | ROV05-02 |
| d__Bacteria;k__norank_d__Bacteria;p__Gemmatimonadetes;c__Gemmatimonadetes;o__Gemmatimonadales;f__Gemmatimonadaceae;g__norank_f__Gemmatimonadaceae;s__uncultured_deep-sea_bacterium_g__norank_f__Gemmatimonadaceae;OTU2121 | ANME-2dI | ROV05-02 |
| d__Bacteria;k__norank_d__Bacteria;p__Gemmatimonadetes;c__Gemmatimonadetes;o__norank_c__Gemmatimonadetes;f__norank_c__Gemmatimonadetes;g__norank_c__Gemmatimonadetes;s__uncultured_bacterium_g__norank_c__Gemmatimonadetes;OTU1750 | ANME-2dI | ROV05-02 |
| d__Bacteria;k__norank_d__Bacteria;p__Gemmatimonadetes;c__Gemmatimonadetes;o__norank_c__Gemmatimonadetes;f__norank_c__Gemmatimonadetes;g__norank_c__Gemmatimonadetes;s__unidentified_bacterium_wb1_A18;OTU1506 | ANME-2dI | ROV05-02 |
| d__Bacteria;k__norank_d__Bacteria;p__Gemmatimonadetes;c__Gemmatimonadetes;o__norank_c__Gemmatimonadetes;f__norank_c__Gemmatimonadetes;g__norank_c__Gemmatimonadetes;s__unidentified_bacterium_wb1_A18;OTU1589 | ANME-2dI | ROV05-02 |
| d__Bacteria;k__norank_d__Bacteria;p__Gemmatimonadetes;c__Gemmatimonadetes;o__norank_c__Gemmatimonadetes;f__norank_c__Gemmatimonadetes;g__norank_c__Gemmatimonadetes;s__unidentified_bacterium_wb1_A18;OTU3858 | ANME-2dI | ROV05-02 |
| d__Bacteria;k__norank_d__Bacteria;p__Kiritimatiellaeota;c__Kiritimatiellae;o__Kiritimatiellales;f__Kiritimatiellaceae;g__R76-B128;s__uncultured_bacterium_g__R76-B128;OTU4750 | ANME-2a/b | ROV05-02 |
| d__Bacteria;k__norank_d__Bacteria;p__Kiritimatiellaeota;c__Kiritimatiellae;o__Kiritimatiellales;f__Kiritimatiellaceae;g__R76-B128;s__uncultured_bacterium_g__R76-B128;OTU4750 | ANME-2a/b | ROV05-02 |
| d__Bacteria;k__norank_d__Bacteria;p__Kiritimatiellaeota;c__Kiritimatiellae;o__Kiritimatiellales;f__Kiritimatiellaceae;g__R76-B128;s__uncultured_bacterium_g__R76-B128;OTU4936 | ANME-2a/b | ROV05-02 |
| d__Bacteria;k__norank_d__Bacteria;p__Kiritimatiellaeota;c__Kiritimatiellae;o__Kiritimatiellales;f__Kiritimatiellaceae;g__R76-B128;s__uncultured_bacterium_g__R76-B128;OTU4936 | ANME-2a/b | ROV05-02 |
| d__Bacteria;k__norank_d__Bacteria;p__Latescibacteria;c__Latescibacteria;o__Latescibacterales;f__Latescibacteraceae;g__norank_f__Latescibacteraceae;s__uncultured_bacterium_g__norank_f__Latescibacteraceae;OTU5162 | ANME-2a/b | ROV07-01 |
| d__Bacteria;k__norank_d__Bacteria;p__Latescibacteria;c__Latescibacteria;o__Latescibacterales;f__Latescibacteraceae;g__norank_f__Latescibacteraceae;s__uncultured_bacterium_g__norank_f__Latescibacteraceae;OTU679 | ANME-2a/b | ROV07-01 |
| d__Bacteria;k__norank_d__Bacteria;p__Latescibacteria;c__Latescibacteria;o__norank_c__Latescibacteria;f__norank_c__Latescibacteria;g__norank_c__Latescibacteria;s__unclassified_g__norank_c__Latescibacteria;OTU4822 | ANME-2a/b | ROV05-02 |
| d__Bacteria;k__norank_d__Bacteria;p__Latescibacteria;c__Latescibacteria;o__norank_c__Latescibacteria;f__norank_c__Latescibacteria;g__norank_c__Latescibacteria;s__unclassified_g__norank_c__Latescibacteria;OTU4822 | ANME-2a/b | ROV05-02 |
| d__Bacteria;k__norank_d__Bacteria;p__Latescibacteria;c__Latescibacteria;o__norank_c__Latescibacteria;f__norank_c__Latescibacteria;g__norank_c__Latescibacteria;s__unclassified_g__norank_c__Latescibacteria;OTU4822 | ANME-2a/b | ROV07-01 |
| d__Bacteria;k__norank_d__Bacteria;p__Nitrospirae;c__Thermodesulfovibrionia;o__norank_c__Thermodesulfovibrionia;f__norank_c__Thermodesulfovibrionia;g__norank_c__Thermodesulfovibrionia;s__marine_sediment_metagenome_g__norank;OTU412 | ANME-2dI | ROV05-02 |
| d__Bacteria;k__norank_d__Bacteria;p__Nitrospirae;c__Thermodesulfovibrionia;o__norank_c__Thermodesulfovibrionia;f__norank_c__Thermodesulfovibrionia;g__norank_c__Thermodesulfovibrionia;s__marine_sediment_metagenome_g__norank;OTU9817 | ANME-2dI | ROV05-02 |
| d__Bacteria;k__norank_d__Bacteria;p__Nitrospirae;c__Thermodesulfovibrionia;o__norank_c__Thermodesulfovibrionia;f__norank_c__Thermodesulfovibrionia;g__norank_c__Thermodesulfovibrionia;s__unclassified_g__norank_c__Thermodesulfovibrionia;OTU2757 | ANME-2dI | ROV05-02 |
| d__Bacteria;k__norank_d__Bacteria;p__Nitrospirae;c__Thermodesulfovibrionia;o__norank_c__Thermodesulfovibrionia;f__norank_c__Thermodesulfovibrionia;g__norank_c__Thermodesulfovibrionia;s__uncultured_bacterium_g__norank_c__Thermodesulfovibrionia;OTU2013 | ANME-2dI | ROV05-02 |
| d__Bacteria;k__norank_d__Bacteria;p__Nitrospirae;c__Thermodesulfovibrionia;o__norank_c__Thermodesulfovibrionia;f__norank_c__Thermodesulfovibrionia;g__norank_c__Thermodesulfovibrionia;s__uncultured_bacterium_g__norank_c__Thermodesulfovibrionia;OTU6099 | ANME-2dI | ROV05-02 |
| d__Bacteria;k__norank_d__Bacteria;p__Patescibacteria;c__Gracilibacteria;o__Candidatus_Peregrinibacteria;f__norank_o__Candidatus_Peregrinibacteria;g__norank_o__Candidatus_Peregrinibacteria;s__unclassified_g__norank_o__Candidatus_Peregrinibacteria;OTU4805 | ANME-2a/b | ROV05-02 |
| d__Bacteria;k__norank_d__Bacteria;p__Patescibacteria;c__Gracilibacteria;o__Candidatus_Peregrinibacteria;f__norank_o__Candidatus_Peregrinibacteria;g__norank_o__Candidatus_Peregrinibacteria;s__unclassified_g__norank_o__Candidatus_Peregrinibacteria;OTU4805 | ANME-2a/b | ROV05-02 |
| d__Bacteria;k__norank_d__Bacteria;p__Patescibacteria;c__Gracilibacteria;o__Candidatus_Peregrinibacteria;f__norank_o__Candidatus_Peregrinibacteria;g__norank_o__Candidatus_Peregrinibacteria;s__uncultured_bacterium_g__norank_o__Candidatus_Peregrinibacteria;OTU293 | ANME-2a/b | ROV05-02 |
| d__Bacteria;k__norank_d__Bacteria;p__Patescibacteria;c__Gracilibacteria;o__Candidatus_Peregrinibacteria;f__norank_o__Candidatus_Peregrinibacteria;g__norank_o__Candidatus_Peregrinibacteria;s__uncultured_bacterium_g__norank_o__Candidatus_Peregrinibacteria;OTU293 | ANME-2a/b | ROV05-02 |
| d__Bacteria;k__norank_d__Bacteria;p__Patescibacteria;c__Gracilibacteria;o__norank_c__Gracilibacteria;f__norank_c__Gracilibacteria;g__norank_c__Gracilibacteria;s__uncultured_sediment_bacterium_g__norank_c__Gracilibacteria;OTU9940 | ANME-2a/b | ROV07-01 |
| d__Bacteria;k__norank_d__Bacteria;p__Patescibacteria;c__Microgenomatia;o__Candidatus_Woesebacteria;f__norank_o__Candidatus_Woesebacteria;g__norank_o__Candidatus_Woesebacteria;s__uncultured_bacterium_g__norank_o__Candidatus_Woesebacteria;OTU10170 | ANME-2a/b | ROV07-01 |
| d__Bacteria;k__norank_d__Bacteria;p__Patescibacteria;c__Microgenomatia;o__Candidatus_Woesebacteria;f__norank_o__Candidatus_Woesebacteria;g__norank_o__Candidatus_Woesebacteria;s__uncultured_bacterium_g__norank_o__Candidatus_Woesebacteria;OTU10222 | ANME-2a/b | ROV07-01 |
| d__Bacteria;k__norank_d__Bacteria;p__Planctomycetes;c__Phycisphaerae;o__Phycisphaerales;f__Phycisphaeraceae;g__Urania-1B-19_marine_sediment_group;s__unclassified_g__Urania-1B-19_marine_sediment_group;OTU9047 | ANME-2dI | ROV05-02 |
| d__Bacteria;k__norank_d__Bacteria;p__Proteobacteria;c__Alphaproteobacteria;o__norank_c__Alphaproteobacteria;f__norank_c__Alphaproteobacteria;g__norank_c__Alphaproteobacteria;s__unclassified_g__norank_c__Alphaproteobacteria;OTU2292 | ANME-2dI | ROV05-02 |
| d__Bacteria;k__norank_d__Bacteria;p__Proteobacteria;c__Alphaproteobacteria;o__Rhizobiales;f__Methyloligellaceae;g__norank_f__Methyloligellaceae;s__unclassified_g__norank_f__Methyloligellaceae;OTU10493 | ANME-2dI | ROV05-02 |
| d__Bacteria;k__norank_d__Bacteria;p__Proteobacteria;c__Alphaproteobacteria;o__Rhizobiales;f__Methyloligellaceae;g__norank_f__Methyloligellaceae;s__unclassified_g__norank_f__Methyloligellaceae;OTU6055 | ANME-2dI | ROV05-02 |
| d__Bacteria;k__norank_d__Bacteria;p__Proteobacteria;c__Alphaproteobacteria;o__Rhizobiales;f__Methyloligellaceae;g__norank_f__Methyloligellaceae;s__uncultured_bacterium_g__norank_f__Methyloligellaceae;OTU10248 | ANME-2dI | ROV05-02 |
| d__Bacteria;k__norank_d__Bacteria;p__Proteobacteria;c__Alphaproteobacteria;o__Rhizobiales;f__Rhizobiaceae;g__Pseudahrensia;s__uncultured_bacterium_g__Pseudahrensia;OTU7797 | ANME-2a/b | ROV05-02 |
| d__Bacteria;k__norank_d__Bacteria;p__Proteobacteria;c__Alphaproteobacteria;o__Rhizobiales;f__Rhizobiaceae;g__Pseudahrensia;s__uncultured_bacterium_g__Pseudahrensia;OTU7797 | ANME-2a/b | ROV05-02 |
| d__Bacteria;k__norank_d__Bacteria;p__Proteobacteria;c__Alphaproteobacteria;o__Rhodobacterales;f__Rhodobacteraceae;g__unclassified_f__Rhodobacteraceae;s__unclassified_f__Rhodobacteraceae;OTU4689 | ANME-2a/b | ROV05-02 |
| d__Bacteria;k__norank_d__Bacteria;p__Proteobacteria;c__Alphaproteobacteria;o__Rhodobacterales;f__Rhodobacteraceae;g__unclassified_f__Rhodobacteraceae;s__unclassified_f__Rhodobacteraceae;OTU4689 | ANME-2a/b | ROV05-02 |
| d__Bacteria;k__norank_d__Bacteria;p__Proteobacteria;c__Alphaproteobacteria;o__Rhodovibrionales;f__Dadabacteriales;g__norank_f__Kiloniellaceae;s__unclassified_g__norank_f__Kiloniellaceae;OTU2074 | ANME-2dI | ROV05-02 |
| d__Bacteria;k__norank_d__Bacteria;p__Proteobacteria;c__Alphaproteobacteria;o__Rhodovibrionales;f__Kiloniellaceae;g__norank_f__Kiloniellaceae;s__uncultured_deep-sea_bacterium_g__norank_f__Kiloniellaceae;OTU2270 | ANME-2dI | ROV05-02 |
| d__Bacteria;k__norank_d__Bacteria;p__Proteobacteria;c__Alphaproteobacteria;o__Sneathiellales;f__Sneathiellaceae;g__AT-s3-44;s__uncultured_deep-sea_bacterium_g__AT-s3-44;OTU2745 | ANME-2dI | ROV05-02 |
| d__Bacteria;k__norank_d__Bacteria;p__Proteobacteria;c__Alphaproteobacteria;o__unclassified_c__Alphaproteobacteria;f__unclassified_c__Alphaproteobacteria;g__unclassified_c__Alphaproteobacteria;s__unclassified_c__Alphaproteobacteria;OTU2001 | ANME-2dI | ROV05-02 |
| d__Bacteria;k__norank_d__Bacteria;p__Proteobacteria;c__Alphaproteobacteria;o__unclassified_c__Alphaproteobacteria;f__unclassified_c__Alphaproteobacteria;g__unclassified_c__Alphaproteobacteria;s__unclassified_c__Alphaproteobacteria;OTU2251 | ANME-2dI | ROV05-02 |
| d__Bacteria;k__norank_d__Bacteria;p__Proteobacteria;c__Alphaproteobacteria;o__unclassified_c__Alphaproteobacteria;f__unclassified_c__Alphaproteobacteria;g__unclassified_c__Alphaproteobacteria;s__unclassified_c__Alphaproteobacteria;OTU9315 | ANME-2dI | ROV05-02 |
| d__Bacteria;k__norank_d__Bacteria;p__Proteobacteria;c__Deltaproteobacteria;o__Deltaproteobacteria_Incertae_Sedis;f__unclassified_o__Deltaproteobacteria_Incertae_Sedis;g__Deferrisoma;s__uncultured_bacterium_g__Deferrisoma;OTU4738 | ANME-2a/b | ROV05-02 |
| d__Bacteria;k__norank_d__Bacteria;p__Proteobacteria;c__Deltaproteobacteria;o__Deltaproteobacteria_Incertae_Sedis;f__unclassified_o__Deltaproteobacteria_Incertae_Sedis;g__Deferrisoma;s__uncultured_bacterium_g__Deferrisoma;OTU4738 | ANME-2a/b | ROV05-02 |
| d__Bacteria;k__norank_d__Bacteria;p__Proteobacteria;c__Deltaproteobacteria;o__Desulfarculales;f__Desulfarculaceae;g__Desulfatiglans;s__unclassified_g__Desulfatiglans;OTU4068 | ANME-2dI | ROV05-02 |
| d__Bacteria;k__norank_d__Bacteria;p__Proteobacteria;c__Deltaproteobacteria;o__Desulfarculales;f__Desulfarculaceae;g__Desulfatiglans;s__uncultured_delta_proteobacterium_g__Desulfatiglans;OTU4333 | ANME-2dI | ROV05-02 |
| d__Bacteria;k__norank_d__Bacteria;p__Proteobacteria;c__Deltaproteobacteria;o__Desulfarculales;f__Desulfarculaceae;g__Desulfatiglans;s__uncultured_Desulfarculaceae_bacterium;OTU7947 | ANME-2a/b | ROV07-01 |
| d__Bacteria;k__norank_d__Bacteria;p__Proteobacteria;c__Deltaproteobacteria;o__Desulfobacterales;f__Desulfobacteraceae;g__Desulfobacterium;s__unclassified_g__Desulfobacterium;OTU8296 | ANME-2a/b | ROV07-01 |
| d__Bacteria;k__norank_d__Bacteria;p__Proteobacteria;c__Deltaproteobacteria;o__Desulfobacterales;f__Desulfobacteraceae;g__norank_f__Desulfobacteraceae;s__unclassified_g__norank_f__Desulfobacteraceae;OTU4638 | ANME-2a/b | ROV05-02 |
| d__Bacteria;k__norank_d__Bacteria;p__Proteobacteria;c__Deltaproteobacteria;o__Desulfobacterales;f__Desulfobacteraceae;g__norank_f__Desulfobacteraceae;s__unclassified_g__norank_f__Desulfobacteraceae;OTU4638 | ANME-2a/b | ROV05-02 |
| d__Bacteria;k__norank_d__Bacteria;p__Proteobacteria;c__Deltaproteobacteria;o__Desulfobacterales;f__Desulfobacteraceae;g__norank_f__Desulfobacteraceae;s__unclassified_g__norank_f__Desulfobacteraceae;OTU7924 | ANME-2a/b | ROV07-01 |
| d__Bacteria;k__norank_d__Bacteria;p__Proteobacteria;c__Deltaproteobacteria;o__Desulfobacterales;f__Desulfobacteraceae;g__norank_f__Desulfobacteraceae;s__unclassified_g__norank_f__Desulfobacteraceae;OTU8102 | ANME-2a/b | ROV05-02 |
| d__Bacteria;k__norank_d__Bacteria;p__Proteobacteria;c__Deltaproteobacteria;o__Desulfobacterales;f__Desulfobacteraceae;g__norank_f__Desulfobacteraceae;s__unclassified_g__norank_f__Desulfobacteraceae;OTU8102 | ANME-2a/b | ROV05-02 |
| d__Bacteria;k__norank_d__Bacteria;p__Proteobacteria;c__Deltaproteobacteria;o__Desulfobacterales;f__Desulfobacteraceae;g__norank_f__Desulfobacteraceae;s__unclassified_g__norank_f__Desulfobacteraceae;OTU8102 | ANME-2a/b | ROV07-01 |
| d__Bacteria;k__norank_d__Bacteria;p__Proteobacteria;c__Deltaproteobacteria;o__Desulfobacterales;f__Desulfobacteraceae;g__norank_f__Desulfobacteraceae;s__uncultured_bacterium_g__norank_f__Desulfobacteraceae;OTU6386 | ANME-2a/b | ROV07-01 |
| d__Bacteria;k__norank_d__Bacteria;p__Proteobacteria;c__Deltaproteobacteria;o__Desulfobacterales;f__Desulfobacteraceae;g__SEEP-SRB1;s__unclassified_g__SEEP-SRB1;OTU754 | ANME-2a/b | ROV05-02 |
| d__Bacteria;k__norank_d__Bacteria;p__Proteobacteria;c__Deltaproteobacteria;o__Desulfobacterales;f__Desulfobacteraceae;g__SEEP-SRB1;s__unclassified_g__SEEP-SRB1;OTU754 | ANME-2a/b | ROV05-02 |
| d__Bacteria;k__norank_d__Bacteria;p__Proteobacteria;c__Deltaproteobacteria;o__Desulfobacterales;f__Desulfobacteraceae;g__Sva0081_sediment_group;s__uncultured_bacterium_g__Sva0081_sediment_group;OTU10181 | ANME-2dI | ROV05-02 |
| d__Bacteria;k__norank_d__Bacteria;p__Proteobacteria;c__Deltaproteobacteria;o__Desulfobacterales;f__Desulfobacteraceae;g__Sva0081_sediment_group;s__uncultured_bacterium_g__Sva0081_sediment_group;OTU3494 | ANME-2dI | ROV05-02 |
| d__Bacteria;k__norank_d__Bacteria;p__Proteobacteria;c__Deltaproteobacteria;o__Desulfobacterales;f__Desulfobacteraceae;g__unclassified_f__Desulfobacteraceae;s__unclassified_f__Desulfobacteraceae;OTU1947 | ANME-2dI | ROV05-02 |
| d__Bacteria;k__norank_d__Bacteria;p__Proteobacteria;c__Deltaproteobacteria;o__Desulfobacterales;f__Desulfobulbaceae;g__Desulfobulbus;s__uncultured_Desulfobulbaceae_bacterium_g__Desulfobulbus;OTU5225 | ANME-2a/b | ROV07-01 |
| d__Bacteria;k__norank_d__Bacteria;p__Proteobacteria;c__Deltaproteobacteria;o__Desulfobacterales;f__Desulfobulbaceae;g__Desulfocapsa;s__unclassified_g__Desulfocapsa;OTU5236 | ANME-2a/b | ROV05-02 |
| d__Bacteria;k__norank_d__Bacteria;p__Proteobacteria;c__Deltaproteobacteria;o__Desulfobacterales;f__Desulfobulbaceae;g__Desulfocapsa;s__unclassified_g__Desulfocapsa;OTU5236 | ANME-2a/b | ROV05-02 |
| d__Bacteria;k__norank_d__Bacteria;p__Proteobacteria;c__Deltaproteobacteria;o__Desulfobacterales;f__Desulfobulbaceae;g__Desulfocapsa;s__uncultured_Desulfobulbaceae_bacterium_g__Desulfocapsa;OTU4838 | ANME-2a/b | ROV05-02 |
| d__Bacteria;k__norank_d__Bacteria;p__Proteobacteria;c__Deltaproteobacteria;o__Desulfobacterales;f__Desulfobulbaceae;g__Desulfocapsa;s__uncultured_Desulfobulbaceae_bacterium_g__Desulfocapsa;OTU4838 | ANME-2a/b | ROV05-02 |
| d__Bacteria;k__norank_d__Bacteria;p__Proteobacteria;c__Deltaproteobacteria;o__Desulfobacterales;f__Desulfobulbaceae;g__Desulfocapsa;s__uncultured_Desulfobulbaceae_bacterium_g__Desulfocapsa;OTU504 | ANME-2a/b | ROV05-02 |
| d__Bacteria;k__norank_d__Bacteria;p__Proteobacteria;c__Deltaproteobacteria;o__Desulfobacterales;f__Desulfobulbaceae;g__Desulfocapsa;s__uncultured_Desulfobulbaceae_bacterium_g__Desulfocapsa;OTU504 | ANME-2a/b | ROV05-02 |
| d__Bacteria;k__norank_d__Bacteria;p__Proteobacteria;c__Deltaproteobacteria;o__Desulfobacterales;f__Desulfobulbaceae;g__norank_f__Desulfobulbaceae;s__unclassified_g__norank_f__Desulfobulbaceae;OTU1800 | ANME-2dI | ROV05-02 |
| d__Bacteria;k__norank_d__Bacteria;p__Proteobacteria;c__Deltaproteobacteria;o__Desulfobacterales;f__Desulfobulbaceae;g__norank_f__Desulfobulbaceae;s__unclassified_g__norank_f__Desulfobulbaceae;OTU6733 | ANME-2dI | ROV05-02 |
| d__Bacteria;k__norank_d__Bacteria;p__Proteobacteria;c__Deltaproteobacteria;o__Desulfobacterales;f__Desulfobulbaceae;g__SEEP-SRB4;s__uncultured_delta_proteobacterium_g__SEEP-SRB4;OTU8128 | ANME-2a/b | ROV05-02 |
| d__Bacteria;k__norank_d__Bacteria;p__Proteobacteria;c__Deltaproteobacteria;o__Desulfobacterales;f__Desulfobulbaceae;g__SEEP-SRB4;s__uncultured_delta_proteobacterium_g__SEEP-SRB4;OTU8128 | ANME-2a/b | ROV05-02 |
| d__Bacteria;k__norank_d__Bacteria;p__Proteobacteria;c__Deltaproteobacteria;o__Desulfobacterales;f__Desulfobulbaceae;g__SEEP-SRB4;s__uncultured_delta_proteobacterium_g__SEEP-SRB4;OTU8128 | ANME-2a/b | ROV07-01 |
| d__Bacteria;k__norank_d__Bacteria;p__Proteobacteria;c__Deltaproteobacteria;o__Desulfuromonadales;f__Desulfuromonadaceae;g__Desulfuromusa;s__unclassified_g__Desulfuromusa;OTU7602 | ANME-2a/b | ROV07-01 |
| d__Bacteria;k__norank_d__Bacteria;p__Proteobacteria;c__Deltaproteobacteria;o__Desulfuromonadales;f__Sva1033;g__norank_f__Sva1033;s__uncultured_bacterium_g__norank_f__Sva1033;OTU4783 | ANME-2a/b | ROV05-02 |
| d__Bacteria;k__norank_d__Bacteria;p__Proteobacteria;c__Deltaproteobacteria;o__Desulfuromonadales;f__Sva1033;g__norank_f__Sva1033;s__uncultured_bacterium_g__norank_f__Sva1033;OTU4783 | ANME-2a/b | ROV05-02 |
| d__Bacteria;k__norank_d__Bacteria;p__Proteobacteria;c__Deltaproteobacteria;o__DTB120;f__norank_o__DTB120;g__norank_o__DTB120;s__unclassified_g__norank_o__DTB120;OTU2929 | ANME-2dI | ROV05-02 |
| d__Bacteria;k__norank_d__Bacteria;p__Proteobacteria;c__Deltaproteobacteria;o__DTB120;f__norank_o__DTB120;g__norank_o__DTB120;s__uncultured_bacterium_g__norank_o__DTB120;OTU2099 | ANME-2dI | ROV05-02 |
| d__Bacteria;k__norank_d__Bacteria;p__Proteobacteria;c__Deltaproteobacteria;o__DTB120;f__norank_o__DTB120;g__norank_o__DTB120;s__uncultured_delta_proteobacterium_g__norank_o__DTB120;OTU2801 | ANME-2dI | ROV05-02 |
| d__Bacteria;k__norank_d__Bacteria;p__Proteobacteria;c__Deltaproteobacteria;o__NB1-j;f__norank_o__NB1-j;g__norank_o__NB1-j;s__uncultured_deep-sea_bacterium_g__norank_o__NB1-j;OTU3540 | ANME-2dI | ROV05-02 |
| d__Bacteria;k__norank_d__Bacteria;p__Proteobacteria;c__Deltaproteobacteria;o__SAR324_cladeMarine_group_B;f__norank_o__SAR324_cladeMarine_group_B;g__norank_o__SAR324_cladeMarine_group_B;s__unclassified_g__norank_o__SAR324_cladeMarine_group_B;OTU1356 | ANME-2dI | ROV05-02 |
| d__Bacteria;k__norank_d__Bacteria;p__Proteobacteria;c__Deltaproteobacteria;o__Syntrophobacterales;f__Syntrophobacteraceae;g__norank_f__Syntrophobacteraceae;s__uncultured_delta_proteobacterium_g__norank_f__Syntrophobacteraceae;OTU10336 | ANME-2dI | ROV05-02 |
| d__Bacteria;k__norank_d__Bacteria;p__Proteobacteria;c__Gammaproteobacteria;o__Cellvibrionales;f__Halieaceae;g__unclassified_f__Halieaceae;s__unclassified_f__Halieaceae;OTU9216 | ANME-2a/b | ROV07-01 |
| d__Bacteria;k__norank_d__Bacteria;p__Proteobacteria;c__Gammaproteobacteria;o__Chromatiales;f__Sedimenticolaceae;g__norank_f__Sedimenticolaceae;s__uncultured_marine_bacterium_g__norank_f__Sedimenticolaceae;OTU4932 | ANME-2a/b | ROV07-01 |
| d__Bacteria;k__norank_d__Bacteria;p__Proteobacteria;c__Gammaproteobacteria;o__Enterobacteriales;f__Enterobacteriaceae;g__Escherichia-Shigella;s__Escherichia_coli_g__Escherichia-Shigella;OTU1599 | ANME-2dI | ROV05-02 |
| d__Bacteria;k__norank_d__Bacteria;p__Proteobacteria;c__Gammaproteobacteria;o__Gammaproteobacteria_Incertae_Sedis;f__unclassified_o__Gammaproteobacteria_Incertae_Sedis;g__norank_f__unclassified_o__Gammaproteobacteria_Incertae_Sedis;s__unclassified_g__norank_f__unclassified_o__Gammaproteobacteria_Incertae_Sedis;OTU5052 | ANME-2a/b | ROV07-01 |
| d__Bacteria;k__norank_d__Bacteria;p__Proteobacteria;c__Gammaproteobacteria;o__Gammaproteobacteria_Incertae_Sedis;f__unclassified_o__Gammaproteobacteria_Incertae_Sedis;g__norank_f__unclassified_o__Gammaproteobacteria_Incertae_Sedis;s__uncultured_proteobacterium_g__norank_f__unclassified;OTU4682 | ANME-2a/b | ROV05-02 |
| d__Bacteria;k__norank_d__Bacteria;p__Proteobacteria;c__Gammaproteobacteria;o__Gammaproteobacteria_Incertae_Sedis;f__unclassified_o__Gammaproteobacteria_Incertae_Sedis;g__norank_f__unclassified_o__Gammaproteobacteria_Incertae_Sedis;s__uncultured_proteobacterium_g__norank_f__unclassified;OTU5143 | ANME-2a/b | ROV05-02 |
| d__Bacteria;k__norank_d__Bacteria;p__Proteobacteria;c__Gammaproteobacteria;o__Gammaproteobacteria_Incertae_Sedis;f__unclassified_o__Gammaproteobacteria_Incertae_Sedis;g__norank_f__unclassified_o__Gammaproteobacteria_Incertae_Sedis;s__uncultured_proteobacterium_g__norank_f__unclassified;OTU4682 | ANME-2a/b | ROV05-02 |
| d__Bacteria;k__norank_d__Bacteria;p__Proteobacteria;c__Gammaproteobacteria;o__Gammaproteobacteria_Incertae_Sedis;f__unclassified_o__Gammaproteobacteria_Incertae_Sedis;g__norank_f__unclassified_o__Gammaproteobacteria_Incertae_Sedis;s__uncultured_proteobacterium_g__norank_f__unclassified;OTU5143 | ANME-2a/b | ROV05-02 |
| d__Bacteria;k__norank_d__Bacteria;p__Proteobacteria;c__Gammaproteobacteria;o__Gammaproteobacteria_Incertae_Sedis;f__unclassified_o__Gammaproteobacteria_Incertae_Sedis;g__norank_f__unclassified_o__Gammaproteobacteria_Incertae_Sedis;s__uncultured_proteobacterium_g__norank_f__unclassified;OTU5143 | ANME-2a/b | ROV07-01 |
| d__Bacteria;k__norank_d__Bacteria;p__Proteobacteria;c__Gammaproteobacteria;o__Gammaproteobacteria_Incertae_Sedis;f__unclassified_o__Gammaproteobacteria_Incertae_Sedis;g__norank_f__unclassified_o__Gammaproteobacteria_Incertae_Sedis;s__uncultured_proteobacterium_g__norank_f__unclassified;OTU4682 | ANME-2a/b | ROV07-01 |
| d__Bacteria;k__norank_d__Bacteria;p__Proteobacteria;c__Gammaproteobacteria;o__Milano-WF1B-44;f__norank_o__Milano-WF1B-44;g__norank_o__Milano-WF1B-44;s__uncultured_bacterium_g__norank_o__Milano-WF1B-44;OTU8047 | ANME-2a/b | ROV07-01 |
| d__Bacteria;k__norank_d__Bacteria;p__Proteobacteria;c__Gammaproteobacteria;o__Steroidobacterales;f__Woeseiaceae;g__Woeseia;s__unclassified_g__Woeseia;OTU4241 | ANME-2a/b | ROV05-02 |
| d__Bacteria;k__norank_d__Bacteria;p__Proteobacteria;c__Gammaproteobacteria;o__Steroidobacterales;f__Woeseiaceae;g__Woeseia;s__unclassified_g__Woeseia;OTU4241 | ANME-2a/b | ROV05-02 |
| d__Bacteria;k__norank_d__Bacteria;p__Proteobacteria;c__Gammaproteobacteria;o__Steroidobacterales;f__Woeseiaceae;g__Woeseia;s__uncultured_deep-sea_bacterium_g__Woeseia;OTU9225 | ANME-2dI | ROV05-02 |
| d__Bacteria;k__norank_d__Bacteria;p__Proteobacteria;c__Gammaproteobacteria;o__Thiomicrospirales;f__Thiomicrospiraceae;g__endosymbionts;s__Maorithyas_hadalis_gill_thioautotrophic_symbiont_II;OTU4982 | ANME-2a/b | ROV05-02 |
| d__Bacteria;k__norank_d__Bacteria;p__Proteobacteria;c__Gammaproteobacteria;o__Thiomicrospirales;f__Thiomicrospiraceae;g__endosymbionts;s__Maorithyas_hadalis_gill_thioautotrophic_symbiont_II;OTU4982 | ANME-2a/b | ROV05-02 |
| d__Bacteria;k__norank_d__Bacteria;p__Proteobacteria;c__Gammaproteobacteria;o__Thiomicrospirales;f__Thiomicrospiraceae;g__endosymbionts;s__Maorithyas_hadalis_gill_thioautotrophic_symbiont_II;OTU4982 | ANME-2a/b | ROV07-01 |
| d__Bacteria;k__norank_d__Bacteria;p__Proteobacteria;c__Gammaproteobacteria;o__Thiomicrospirales;f__Thiomicrospiraceae;g__endosymbionts;s__unclassified_g__endosymbionts;OTU8145 | ANME-2a/b | ROV07-01 |
| d__Bacteria;k__norank_d__Bacteria;p__Proteobacteria;c__Gammaproteobacteria;o__Thiotrichales;f__Thiotrichaceae;g__Cocleimonas;s__uncultured_bacterium_g__Cocleimonas;OTU7763 | ANME-2a/b | ROV05-02 |
| d__Bacteria;k__norank_d__Bacteria;p__Proteobacteria;c__Gammaproteobacteria;o__Thiotrichales;f__Thiotrichaceae;g__Cocleimonas;s__uncultured_bacterium_g__Cocleimonas;OTU7763 | ANME-2a/b | ROV05-02 |
| d__Bacteria;k__norank_d__Bacteria;p__Proteobacteria;c__Gammaproteobacteria;o__Thiotrichales;f__Thiotrichaceae;g__norank_f__Thiotrichaceae;s__uncultured_hydrocarbon_seep_bacterium_BPC036;OTU10075 | ANME-2a/b | ROV05-02 |
| d__Bacteria;k__norank_d__Bacteria;p__Proteobacteria;c__Gammaproteobacteria;o__Thiotrichales;f__Thiotrichaceae;g__norank_f__Thiotrichaceae;s__uncultured_hydrocarbon_seep_bacterium_BPC036;OTU10075 | ANME-2a/b | ROV05-02 |
| d__Bacteria;k__norank_d__Bacteria;p__Proteobacteria;c__Gammaproteobacteria;o__Thiotrichales;f__Thiotrichaceae;g__norank_f__Thiotrichaceae;s__uncultured_sediment_bacterium_g__norank_f__Thiotrichaceae;OTU4672 | ANME-2a/b | ROV05-02 |
| d__Bacteria;k__norank_d__Bacteria;p__Proteobacteria;c__Gammaproteobacteria;o__Thiotrichales;f__Thiotrichaceae;g__norank_f__Thiotrichaceae;s__uncultured_sediment_bacterium_g__norank_f__Thiotrichaceae;OTU4672 | ANME-2a/b | ROV05-02 |
| d__Bacteria;k__norank_d__Bacteria;p__Proteobacteria;c__Gammaproteobacteria;o__Thiotrichales;f__Thiotrichaceae;g__norank_f__Thiotrichaceae;s__uncultured_sediment_bacterium_g__norank_f__Thiotrichaceae;OTU5287 | ANME-2a/b | ROV05-02 |
| d__Bacteria;k__norank_d__Bacteria;p__Proteobacteria;c__Gammaproteobacteria;o__Thiotrichales;f__Thiotrichaceae;g__norank_f__Thiotrichaceae;s__uncultured_sediment_bacterium_g__norank_f__Thiotrichaceae;OTU5287 | ANME-2a/b | ROV05-02 |
| d__Bacteria;k__norank_d__Bacteria;p__Proteobacteria;c__Gammaproteobacteria;o__Thiotrichales;f__Thiotrichaceae;g__norank_f__Thiotrichaceae;s__uncultured_sediment_bacterium_g__norank_f__Thiotrichaceae;OTU5374 | ANME-2a/b | ROV07-01 |
| d__Bacteria;k__norank_d__Bacteria;p__Proteobacteria;c__Gammaproteobacteria;o__unclassified_c__Gammaproteobacteria;f__unclassified_c__Gammaproteobacteria;g__unclassified_c__Gammaproteobacteria;s__unclassified_c__Gammaproteobacteria;OTU9195 | ANME-2dI | ROV05-02 |
| d__Bacteria;k__norank_d__Bacteria;p__unclassified_k__norank_d__Bacteria;c__unclassified_k__norank_d__Bacteria;o__unclassified_k__norank_d__Bacteria;f__unclassified_k__norank_d__Bacteria;g__unclassified_k__norank_d__Bacteria;s__unclassified_k__norank_d__Bacteria;OTU5678 | ANME-2a/b | ROV07-01 |

# References

Friedrich, M.W. (2002). Phylogenetic analysis reveals multiple lateral transfers of adenosine-5′-phosphosulfate reductase genes among sulfate-reducing microorganisms. *Journal of bacteriology* 184(1), 278-289.

Lloyd, K.G., Alperin, M.J., and Teske, A. (2011). Environmental evidence for net methane production and oxidation in putative ANaerobic MEthanotrophic (ANME) archaea. *Environmental Microbiology* 13(9), 2548-2564.

Luton, P.E., Wayne, J.M., Sharp, R.J., and Riley, P.W. (2002). The mcrA gene as an alternative to 16S rRNA in the phylogenetic analysis of methanogen populations in landfillb. *Microbiology* 148(11), 3521-3530.

Mori, H., Maruyama, F., Kato, H., Toyoda, A., Dozono, A., Ohtsubo, Y., et al. (2013). Design and experimental application of a novel non-degenerate universal primer set that amplifies prokaryotic 16S rRNA genes with a low possibility to amplify eukaryotic rRNA genes. *DNA research* 21(2), 217-227.

Nadkarni, M.A., Martin, F.E., Jacques, N.A., and Hunter, N. (2002). Determination of bacterial load by real-time PCR using a broad-range (universal) probe and primers set. *Microbiology* 148(1), 257-266.

Raskin, L., Stromley, J.M., Rittmann, B.E., and Stahl, D.A. (1994). Group-specific 16S rRNA hybridization probes to describe natural communities of methanogens. *Appl. Environ. Microbiol.* 60(4), 1232-1240.

Stahl, D. (1991). Development and application of nucleic acid probes. *Nucleic acid techniques in bacterial systematics*, 205-248.

Vigneron, A., Cruaud, P., Pignet, P., Caprais, J.C., Cambon Bonavita, M.A., Godfroy, A., et al. (2013). Archaeal and anaerobic methane oxidizer communities in the Sonora Margin cold seeps, Guaymas Basin (Gulf of California). *The ISME journal* 7(8), 1595.

Yu, Y., Lee, C., Kim, J., and Hwang, S. (2005). Group‐specific primer and probe sets to detect methanogenic communities using quantitative real‐time polymerase chain reaction. *Biotechnology and bioengineering* 89(6), 670-679.
